# Supplementary material for: Stoic attitude in patients with cancer from the NEOcoping study: Cross-sectional study
Source: PLoS One. 2022 Jul 28;17(7):e0269712. doi: 10.1371/journal.pone.0269712 (PMC9333303; doi:10.1371/journal.pone.0269712)
Supplement: S1 Table — (PDF) [file pone.0269712.s001.pdf]

Supporting Information

Stoic attitude in patients with cancer from the NEOcoping study: cross-sectional study

David Gomez[1], Alberto Carmona-Bayonas[2], Raquel Hernandez[3], Oliver Higuera[4], Jacobo Rogado-Revuelta[5], Vilma Pacheco-Barcia[6], María Valero[7], Mireia Gil Raga[8], Mª Mar Muñoz[9], Rafael Carrión-Galindo[10], Paula Jimenez-Fonseca[11], Caterina Calderon[12]

[1] Department of Medical Oncology. Hospital Universitario Central of Asturias, IPSA, Oviedo, Universidad del País Vasco (UPV/EHU), País Vasco, Spain. https://orcid.org/0000-0002-1930-9660
[2] Department of Hematology and Medical Oncology, Hospital Universitario Morales Meseguer, Instituto Murciano de Investigación Biosanitaria (IMIB), UMU, Murcia, Spain. https://orcid.org/0000-0002-7265-0251
[3] Department of Medical Oncology. Hospital Universitario de Canarias, Tenerife, Spain. https://orcid.org/0000-0003-3426-7515
[4] Department of Medical Oncology. Hospital Universitario La Paz, Madrid, Spain.
[5] Department of Medical Oncology. Hospital Universitario Infanta Leonor, Madrid, Spain. https://orcid.org/0000-0002-9795-8762
[6] Department of Medical Oncology. Hospital Central de la Defensa Gomez Ulla, Madrid, Spain.
[7] Department of Medical Oncology. Hospital Quirón salud Sagrado Corazón, Sevilla, Spain. https://orcid.org/0000-0002-2539-5710
[8] Department of Medical Oncology. Consorcio Hospital General Universitario de Valencia, Valencia, Spain.
[9] Department of Medical Oncology. Hospital Virgen de La Luz, Cuenca, Spain. https://orcid.org/0000-0003-2579-8947
[10] Department of Medical Oncology. Hospital Universitario del Sureste, Arganda del Rey-Madrid, Spain.
[11] Department of Medical Oncology. Hospital Universitario Central of Asturias, IPSA, Oviedo, Universidad del País Vasco (UPV/EHU), País Vasco, Spain. https://orcid.org/0000-0003-4592-3813
[12] Department of Clinical Psychology and Psychobiology. Faculty of Psychology. University of Barcelona, Barcelona, University of País Vasco (UPV/EHU), Pais Vasco, Spain. https://orcid.org/0000-0002-6956-9321

Caterina Calderon, PhD.
Department of Clinical Psychology and Psychobiology. Faculty of Psychology. University of Barcelona, Spain.
Passeig de la Vall d'Hebron, 171. 08035 Barcelona, Spain.
ORCID: 0000-0002-6956-9321
Email: ccalderon@ub.edu

Table S1. The table shows the sex, age and patients scores on psychological scales.

Sex	Age	LSS	MSPSS	LOT	MAC_HH	MAC_AP	MAC_PA	MAC_CA	BSISom	BSIAn	BSIDe
Men	70	73	81	14	52	70	67	17	62	69	69
Men	66	54	73	19	57	63	44	58	66	65	57
Men	48	57	81	24	57	33	81	58	66	64	57
Women	75	41	70	15	76	100	30	25	62	73	73
Men	50	54	76	19	0	10	41	0	66	52	61
Men	68	52	84	28	14	43	85	92	54	61	54
Women	60	54	72	25	29	57	74	50	65	64	65
Men	76	60	63	20	10	33	78	33	57	55	61
Men	45	59	84	30	57	93	100	100	54	52	54
Women	50	50	77	21	10	70	70	50	67	75	67
Men	69	62	69	17	10	7	70	25	54	52	54
Men	70	62	84	22	0	3	70	67	57	52	54
Women	51	51	83	21	0	30	74	50	66	59	54
Men	79	53	68	25	0	57	96	75	64	69	64
Women	68	50	76	6	48	80	70	92	64	72	70
Women	70	70	78	21	14	40	74	67	67	69	64
Men	66	52	80	21	48	53	74	75	54	52	54
Men	79	59	77	21	0	17	59	33	57	52	54
Women	68	55	84	20	19	67	85	58	55	61	54

Men	45	59	84	30	57	93	100	100	54	52	54
Men	46	63	82	15	5	20	56	33	54	52	61
Women	43	58	80	16	29	67	67	67	74	74	70
Men	61	55	84	21	14	17	56	0	57	52	54
Women	74	62	76	17	0	83	52	58	66	52	61
Men	69	61	67	18	0	47	89	100	57	59	57
Men	47	66	84	18	0	17	48	0	54	52	54
Men	51	51	68	12	14	30	56	75	62	61	61
Women	65	58	60	26	0	20	56	50	57	55	54
Women	33	51	72	21	0	10	63	25	54	52	54
Women	36	31	76	28	5	50	81	83	57	64	57
Women	60	41	77	27	0	30	89	42	57	55	57
Women	58	57	53	21	14	47	56	42	78	71	72
Men	61	64	71	26	29	3	93	58	54	52	54
Men	62	59	71	26	10	23	78	67	62	64	64
Women	57	52	80	26	0	23	89	83	54	59	54
Women	50	44	71	15	5	17	67	25	54	55	54
Women	44	50	79	19	0	37	96	17	66	52	54
Women	48	56	84	22	0	20	78	33	54	61	61
Women	46	55	77	23	0	27	85	33	57	64	57
Women	54	66	49	15	38	73	78	50	66	75	72
Men	69	60	74	22	29	27	89	83	54	63	54

Women	38	50	84	25	0	33	85	100	62	59	61
Women	73	49	84	18	57	33	89	83	54	52	54
Women	32	55	63	22	48	90	81	100	57	66	69
Women	50	55	75	15	48	90	59	67	67	80	76
Women	62	58	84	15	43	80	100	92	69	75	69
Women	51	66	80	21	0	20	89	67	68	64	64
Women	43	50	73	17	0	40	85	50	61	61	61
Women	56	51	84	28	0	63	85	75	68	73	65
Women	53	53	78	19	19	50	93	33	54	64	61
Men	66	60	79	19	48	33	85	83	54	59	54
Men	79	72	73	21	5	20	78	75	55	52	54
Women	61	54	79	19	48	87	59	42	62	52	61
Men	62	48	84	26	0	40	93	67	54	52	54
Men	57	56	72	19	29	17	74	17	62	61	54
Women	71	47	84	28	0	47	93	75	54	63	61
Men	65	70	71	18	57	97	63	58	66	80	80
Women	48	45	74	25	29	53	85	92	80	74	65
Men	59	50	75	26	0	3	89	25	54	52	54
Women	47	47	80	24	0	20	85	33	54	55	57
Women	52	54	82	18	0	33	74	42	57	68	63
Men	61	70	74	20	29	27	81	67	57	64	67
Men	56	60	84	17	57	80	70	83	62	61	65

Women	46	51	84	17	10	77	89	92	68	75	68
Men	63	68	77	27	19	40	63	33	54	52	54
Men	52	59	84	23	43	47	100	50	57	65	61
Men	69	61	55	24	5	20	30	17	54	52	61
Women	48	54	78	21	33	17	100	42	57	63	61
Women	53	58	77	23	0	7	89	58	64	55	54
Men	58	60	70	20	29	20	59	58	69	68	69
Men	67	80	72	20	19	23	67	75	74	52	61
Women	63	62	75	15	33	70	74	67	64	65	64
Women	59	56	72	20	29	33	74	33	54	59	61
Women	53	53	84	25	0	13	78	0	54	52	54
Women	51	50	80	19	5	63	89	75	67	69	67
Men	69	49	84	21	19	20	74	67	54	55	54
Men	50	60	77	22	24	20	70	42	68	59	57
Men	67	59	80	12	14	47	81	58	57	66	61
Women	54	66	81	20	38	33	81	50	54	61	64
Women	84	49	81	20	57	53	78	67	66	55	61
Men	73	59	79	17	38	57	74	58	54	59	61
Men	66	74	70	15	29	43	70	42	64	63	61
Women	50	42	78	26	0	27	89	67	65	69	65
Women	59	56	82	28	0	23	81	42	57	59	54
Men	61	58	79	24	38	70	85	75	65	55	65

Men	40	67	52	19	0	47	63	33	54	68	65
Women	68	55	56	18	43	17	19	0	64	66	72
Women	70	68	60	16	14	63	70	67	76	69	70
Men	52	62	70	19	24	80	78	75	62	69	68
Men	55	50	84	27	5	50	81	33	66	61	61
Men	72	63	80	20	0	50	78	50	57	66	63
Women	58	53	70	29	14	27	63	83	67	63	61
Women	47	40	83	28	0	23	74	25	54	59	57
Women	73	53	84	19	43	27	100	100	66	55	61
Women	28	53	69	20	19	57	63	75	78	80	70
Men	74	58	73	16	29	43	59	58	57	64	61
Men	78	56	77	22	24	30	81	75	66	61	54
Women	57	60	76	17	10	40	70	33	54	63	61
Women	43	66	54	10	62	100	81	100	80	80	76
Men	39	71	63	18	5	50	78	83	66	52	68
Women	43	50	73	20	10	47	70	58	54	71	61
Women	50	63	53	20	24	77	93	83	68	63	69
Women	61	63	51	17	0	20	93	92	54	71	68
Men	74	55	76	20	48	33	74	67	54	55	61
Women	28	45	80	25	29	33	56	67	54	65	61
Men	68	60	48	18	33	37	33	33	78	72	71
Women	32	62	84	23	10	73	93	92	67	80	71

Men	65	60	77	20	57	67	26	25	62	52	57
Women	39	64	68	26	5	23	89	75	64	59	61
Men	62	53	84	17	38	73	78	67	78	71	71
Women	48	51	59	14	24	80	56	67	74	80	74
Women	58	58	78	23	14	3	41	0	54	52	54
Women	62	60	62	21	33	33	67	58	64	65	67
Women	48	63	69	22	38	40	67	42	69	73	69
Women	46	51	82	25	0	30	81	50	54	61	57
Women	62	55	80	25	19	23	85	83	57	64	61
Women	59	47	77	23	5	70	85	92	54	65	61
Men	71	71	82	14	29	60	56	50	68	64	68
Men	46	55	80	20	0	50	89	67	74	80	71
Women	48	56	73	20	10	63	78	92	57	71	68
Men	79	65	60	17	19	3	22	0	54	52	54
Men	61	72	69	19	5	53	56	42	67	63	63
Men	62	56	84	28	0	37	81	58	54	59	61
Women	69	64	68	13	29	87	59	58	68	80	70
Women	63	51	80	22	33	40	81	75	66	55	61
Women	50	44	84	19	33	20	78	17	54	59	57
Women	40	48	76	23	0	10	89	42	62	52	61
Women	43	54	54	20	33	63	59	42	54	63	61
Women	51	47	64	21	0	40	85	50	57	66	57

Men	79	57	84	22	14	30	81	50	66	55	54
Women	45	50	84	24	29	43	100	42	66	59	54
Men	70	62	67	21	0	20	56	25	57	59	57
Women	38	37	84	25	0	20	100	75	68	66	57
Women	37	56	84	15	0	33	100	100	54	52	54
Women	40	60	77	19	5	37	81	42	64	59	63
Women	46	40	80	26	5	23	93	58	57	63	57
Men	57	61	76	22	5	30	63	8	62	61	61
Women	52	45	81	19	33	67	89	67	70	75	70
Men	60	66	63	19	5	17	56	42	55	55	54
Women	47	43	84	30	14	30	81	17	54	61	54
Women	43	52	84	18	0	40	81	33	57	55	57
Women	78	57	80	22	29	23	85	67	54	55	57
Men	55	59	74	19	33	57	89	58	62	59	64
Women	49	64	50	10	67	93	30	8	78	80	76
Men	33	66	69	7	52	73	37	75	54	66	73
Women	45	54	77	16	38	73	85	67	66	65	63
Women	63	55	55	22	38	73	89	100	66	69	70
Men	73	60	74	20	10	17	67	42	62	55	54
Women	52	52	84	20	14	50	59	83	62	80	69
Women	61	59	84	21	0	13	70	42	54	55	54
Women	76	65	69	19	29	63	70	58	57	61	63

Women	62	60	80	24	10	27	81	33	54	52	54
Women	58	57	72	17	5	40	70	75	54	64	61
Women	47	59	49	18	10	57	78	67	66	65	65
Women	55	58	50	17	52	20	78	50	57	64	61
Women	69	58	80	19	29	30	100	83	62	55	63
Women	64	60	84	25	19	37	67	8	78	55	61
Women	50	40	84	27	0	20	85	25	54	59	54
Men	69	51	84	23	10	17	56	25	54	52	54
Women	72	59	82	17	33	80	81	100	62	71	67
Women	60	50	84	15	29	60	89	83	68	71	76
Women	72	54	77	21	67	47	78	100	64	63	64
Men	82	63	71	18	43	83	70	50	54	80	64
Men	72	56	71	19	5	17	81	33	54	59	54
Men	79	64	52	20	0	30	33	25	62	55	65
Women	74	48	84	12	0	20	78	33	62	66	65
Men	60	61	30	24	14	40	89	67	54	52	54
Women	59	62	72	19	5	27	63	50	61	61	61
Men	73	50	79	20	5	63	93	75	66	69	68
Women	57	61	80	28	0	17	93	25	61	63	61
Women	49	67	72	18	14	30	33	17	66	71	71
Men	77	53	81	24	29	80	89	67	57	65	57
Men	54	63	82	19	52	53	85	75	54	59	61

Women	71	60	65	18	10	40	81	83	66	63	69
Men	75	54	56	17	43	83	89	83	62	63	54
Women	57	50	74	14	19	37	52	0	64	65	63
Women	45	48	66	24	5	43	70	33	74	65	65
Women	53	54	84	22	62	60	81	92	57	75	65
Women	38	34	84	24	0	70	89	100	63	80	63
Women	50	52	84	28	0	27	78	58	54	63	57
Men	77	56	82	19	48	37	96	100	54	61	61
Women	66	49	84	29	0	13	81	17	54	52	61
Men	67	48	79	21	62	23	93	58	57	59	57
Women	62	61	77	16	38	50	52	25	64	72	65
Women	35	58	73	22	0	37	100	83	66	64	63
Women	74	58	74	23	0	13	56	33	54	63	54
Women	52	47	84	15	24	97	48	8	54	73	67
Women	51	50	81	16	33	90	85	100	62	71	63
Women	71	54	78	23	62	67	89	92	62	71	68
Women	65	60	84	24	14	17	89	67	54	52	54
Men	43	50	84	25	0	27	89	42	66	61	57
Men	63	52	73	23	29	57	81	42	63	52	63
Men	67	60	83	18	14	20	96	100	54	59	54
Men	65	70	41	12	5	87	96	50	80	80	76
Women	57	50	84	22	0	43	89	42	57	55	61

Women	69	67	73	24	0	13	96	67	54	61	57
Women	51	64	62	17	38	80	67	75	64	55	64
Men	75	65	84	19	29	23	48	25	54	59	54
Women	49	51	75	25	5	63	81	67	62	61	61
Women	51	59	79	20	24	3	81	0	62	55	61
Women	46	49	83	19	43	60	100	83	68	72	57
Women	46	40	84	26	5	30	89	42	64	64	57
Women	58	63	83	27	0	20	44	0	55	55	54
Women	50	55	77	27	0	10	96	42	55	55	54
Women	69	77	66	18	14	30	44	58	57	64	65
Women	71	48	84	24	14	20	78	33	57	52	54
Women	40	46	75	21	10	60	70	50	66	66	63
Women	71	50	84	26	19	13	96	92	54	52	54
Women	62	55	81	24	19	50	78	58	54	64	57
Women	44	61	80	17	14	80	85	58	57	55	57
Men	75	54	72	11	5	23	63	25	54	64	63
Women	65	69	84	17	71	13	63	25	66	52	61
Women	59	57	62	22	33	73	67	92	57	68	64
Men	39	50	80	17	33	47	59	25	54	61	57
Men	69	56	68	25	29	23	96	33	54	52	54
Women	64	50	80	22	33	37	93	67	67	63	61
Women	39	35	84	22	0	27	85	58	57	66	61

Men	55	45	76	24	0	33	96	67	57	52	57
Women	71	76	56	13	62	83	70	75	76	80	76
Women	50	50	82	22	0	27	78	33	80	66	61
Women	57	55	84	24	19	17	67	17	57	61	54
Men	44	61	70	23	14	27	78	17	62	55	57
Men	73	60	68	19	5	23	78	92	61	59	61
Women	70	57	66	17	43	70	70	58	67	75	69
Men	68	58	72	19	24	23	67	33	54	52	54
Men	71	58	70	19	52	33	96	67	54	59	54
Women	41	48	81	20	19	53	67	42	66	68	64
Men	78	53	84	22	0	3	100	100	54	55	54
Women	45	46	84	27	24	7	100	83	54	55	54
Women	70	56	56	21	14	30	67	42	66	55	61
Women	44	37	84	15	10	37	81	42	66	65	61
Men	59	54	82	22	33	40	85	83	54	61	54
Men	54	58	74	26	10	73	93	92	78	65	63
Women	76	53	84	19	5	20	78	17	54	55	54
Women	50	53	79	23	0	23	67	25	54	61	57
Men	67	66	57	17	57	30	67	58	76	71	70
Women	46	57	84	28	19	73	85	75	54	65	54
Men	68	67	78	17	43	53	67	58	64	64	64
Men	73	58	70	12	33	73	56	67	74	64	65

Men	56	66	73	20	0	13	63	25	66	63	54
Men	40	57	76	22	52	47	78	33	57	63	61
Women	68	61	78	25	5	87	96	75	76	63	73
Men	56	57	77	16	5	27	63	67	62	61	61
Men	62	62	81	20	0	23	70	25	57	55	54
Women	47	54	82	23	38	47	67	42	54	61	57
Men	66	62	72	21	43	67	81	67	67	69	63
Women	59	66	77	19	67	20	85	50	66	55	63
Men	67	54	76	29	0	23	100	58	57	52	54
Men	72	62	82	21	14	40	59	25	61	52	61
Women	47	46	63	22	24	37	59	33	74	66	65
Men	61	69	78	29	0	70	93	67	78	63	61
Men	63	69	58	18	14	67	100	58	54	71	64
Men	68	71	60	15	67	87	89	83	57	66	68
Women	46	44	77	15	0	23	41	42	74	69	54
Women	70	30	84	30	14	57	93	75	54	55	54
Women	35	48	84	25	0	37	85	67	54	66	54
Men	67	53	77	21	52	33	93	83	57	61	57
Women	50	42	82	25	0	27	44	8	62	64	57
Men	68	56	63	19	19	17	81	50	54	59	57
Women	73	61	64	17	10	43	63	67	57	61	54
Men	76	50	77	22	43	43	81	92	66	63	61

Women	60	60	83	18	38	57	63	50	54	65	71
Women	75	49	76	20	0	10	70	92	62	61	54
Women	51	36	60	29	0	10	89	0	66	52	54
Men	25	73	58	19	33	47	26	0	66	68	70
Women	64	55	84	24	0	23	100	100	54	55	54
Women	69	56	80	17	19	67	52	50	57	65	63
Women	42	58	80	20	0	13	89	8	61	64	61
Women	73	63	68	12	33	97	59	100	66	71	71
Men	61	58	70	16	86	50	100	67	54	55	61
Women	73	51	69	24	48	57	70	50	57	69	65
Women	64	57	84	24	14	37	96	83	54	52	54
Men	77	67	66	18	0	7	67	8	54	52	54
Women	54	46	84	27	19	87	63	50	66	80	72
Women	55	47	80	20	0	27	30	0	64	52	61
Women	71	61	80	24	48	63	22	33	68	61	61
Women	67	62	77	22	0	57	89	100	54	64	61
Women	75	49	71	22	19	83	67	67	62	66	65
Men	80	46	84	30	0	3	78	58	66	52	61
Women	63	60	79	17	0	33	93	83	64	63	61
Men	70	52	83	22	14	47	85	67	64	65	57
Women	65	55	84	20	14	23	93	83	54	61	54
Women	74	55	84	24	43	37	96	100	61	59	61

Women	62	40	84	25	19	80	81	83	66	64	63
Men	60	59	69	22	5	10	70	0	66	59	54
Women	46	49	77	27	0	23	67	42	67	65	61
Women	45	58	72	19	10	53	81	83	67	72	69
Women	47	56	79	18	0	33	78	75	57	68	61
Women	51	47	69	23	5	20	63	17	57	55	57
Men	79	54	84	21	14	10	81	33	64	55	54
Women	53	42	62	14	43	73	26	33	80	80	71
Women	62	64	66	26	0	37	52	33	54	52	54
Women	68	68	72	20	52	83	48	25	61	63	61
Women	43	44	69	20	29	77	74	58	66	73	61
Men	62	53	80	23	43	27	78	42	57	55	54
Women	32	48	84	26	5	37	52	8	64	59	63
Men	75	54	73	23	19	17	85	42	66	61	54
Women	61	36	84	24	5	73	56	33	71	65	71
Women	60	56	69	20	19	60	67	83	54	65	67
Women	49	55	75	29	0	47	100	67	66	59	61
Women	66	60	30	24	0	10	85	75	54	52	54
Women	64	66	78	21	19	40	74	58	57	63	61
Women	52	57	84	19	0	30	89	50	64	64	54
Men	75	60	70	17	14	33	89	33	62	59	54
Men	59	64	68	18	10	20	37	17	57	52	57

Women	54	44	83	22	19	50	81	58	68	59	64
Men	57	61	84	20	0	40	67	58	66	63	61
Women	44	44	84	28	5	40	78	83	54	59	57
Men	62	53	84	25	14	27	85	92	54	55	54
Women	56	52	84	26	38	93	78	100	76	80	80
Men	78	64	68	21	0	33	74	50	54	52	54
Women	57	66	46	12	62	80	52	67	54	74	72
Women	53	50	44	25	10	27	70	25	78	71	68
Women	56	51	79	22	0	7	85	92	62	71	61
Women	49	54	78	27	29	30	85	42	54	65	54
Women	80	53	80	30	0	23	100	67	64	59	54
Men	68	51	83	18	29	60	96	100	64	55	54
Men	57	49	84	26	5	50	93	50	66	69	68
Women	43	54	84	19	48	60	85	42	71	68	71
Men	35	49	84	19	14	37	56	25	54	63	63
Women	69	61	36	26	0	20	63	33	64	55	54
Women	62	48	66	17	5	50	59	33	62	72	67
Women	81	57	82	26	19	57	81	42	68	66	61
Men	34	66	69	7	48	70	37	75	54	66	73
Women	75	52	72	20	24	30	70	42	57	61	54
Women	45	60	79	20	29	73	81	58	57	61	61
Women	71	61	73	19	10	53	70	42	66	66	69

Men	64	48	84	20	0	7	96	42	54	52	54
Women	48	46	80	25	14	27	70	50	66	61	57
Women	52	47	79	27	0	43	63	8	66	66	61
Women	53	65	82	21	0	23	74	33	62	66	61
Women	74	59	51	22	24	63	63	33	57	66	67
Women	49	52	84	28	0	17	100	25	54	55	54
Men	81	60	84	19	48	87	78	58	80	73	71
Men	74	58	84	19	38	63	78	58	64	64	57
Men	48	65	62	11	52	97	78	58	62	72	71
Men	59	53	82	25	5	33	74	83	64	52	63
Men	53	60	77	16	24	50	81	83	64	65	64
Women	36	47	76	22	0	60	89	50	66	73	68
Men	65	59	84	19	24	40	93	58	54	61	57
Men	62	60	80	20	38	77	63	58	62	64	63
Women	54	49	84	19	57	73	78	92	62	63	57
Women	43	39	72	14	33	53	63	58	67	63	54
Men	66	61	75	18	52	50	81	75	67	63	61
Women	57	50	65	23	14	17	67	25	78	61	54
Women	34	39	82	24	0	33	85	25	67	59	63
Women	40	56	78	23	0	7	93	25	62	55	57
Women	45	61	77	13	24	50	70	75	68	68	63
Men	75	47	84	24	0	13	85	42	54	52	54

Women	65	60	64	22	14	33	67	67	74	66	64
Women	57	61	44	22	10	47	74	50	78	80	68
Women	31	57	84	21	5	20	100	33	67	55	54
Women	65	60	30	21	0	17	74	17	54	52	54
Men	26	64	70	16	43	80	56	83	66	74	69
Men	84	69	84	7	0	27	85	33	54	59	54
Men	55	60	71	16	10	23	52	8	64	52	54
Women	63	44	84	16	52	90	52	25	80	72	74
Women	73	48	67	20	14	23	89	75	54	59	61
Women	72	52	75	20	14	73	74	58	54	59	61
Men	41	62	84	20	14	17	89	75	66	52	54
Men	55	54	81	21	0	23	78	33	57	52	61
Men	57	60	43	18	29	43	59	50	64	65	71
Women	58	43	84	26	0	63	67	92	65	71	65
Men	51	63	70	19	24	40	56	25	66	61	63
Men	56	54	74	27	0	10	93	50	67	52	54
Men	59	72	64	18	14	63	93	83	68	72	71
Women	70	44	72	15	0	7	74	92	55	52	54
Women	39	55	77	19	10	67	67	17	62	74	70
Men	59	67	77	29	0	33	63	17	66	65	57
Women	54	53	73	20	29	43	93	75	54	65	67
Women	68	57	71	20	57	40	81	67	61	64	61

Men	36	66	75	14	24	47	41	42	66	66	67
Women	63	51	69	19	0	30	85	33	54	55	57
Men	64	63	58	18	52	20	63	25	57	61	57
Men	70	56	80	25	24	10	70	0	54	52	54
Men	60	54	77	28	14	33	96	50	54	55	57
Women	41	34	77	21	0	50	85	25	66	72	68
Women	38	45	78	26	0	13	78	33	57	61	57
Women	79	68	72	22	0	7	52	42	54	52	54
Women	45	49	83	18	62	87	89	83	54	73	67
Women	62	59	44	22	14	20	59	25	64	61	65
Women	46	49	79	27	0	7	85	25	67	52	54
Men	41	55	59	14	19	67	59	67	57	71	73
Men	54	56	81	22	14	40	63	67	54	66	64
Women	85	56	84	18	10	23	70	50	57	61	61
Women	32	57	83	23	43	53	96	58	57	61	61
Men	69	62	77	20	0	7	26	8	62	52	54
Women	69	58	78	17	0	33	63	67	54	69	61
Women	63	49	84	25	24	17	89	75	54	68	57
Women	33	35	62	21	0	33	74	42	64	59	63
Women	59	60	76	25	14	83	67	42	67	69	63
Women	46	48	84	26	14	13	93	25	61	59	61
Men	57	54	84	25	5	33	89	75	76	71	57

Men	60	48	82	22	5	33	52	83	57	63	54
Women	37	56	72	23	5	53	48	8	62	68	64
Men	56	51	79	23	29	27	78	58	78	59	63
Women	59	59	65	24	0	20	85	67	66	63	64
Men	69	70	83	22	0	27	89	67	66	61	61
Men	44	59	24	19	19	57	67	58	65	68	65
Women	60	57	76	22	19	70	85	83	66	69	64
Men	74	59	78	24	0	27	74	75	62	55	57
Women	61	60	84	20	14	33	44	33	63	64	61
Women	56	52	84	22	19	83	93	75	66	73	61
Women	67	57	84	21	0	37	81	67	57	66	61
Men	72	63	40	20	29	23	48	33	68	61	61
Women	71	59	80	16	19	37	81	58	62	59	54
Men	64	48	84	23	19	37	93	92	57	55	54
Women	70	52	70	22	14	33	89	25	54	55	57
Women	61	69	79	27	0	23	81	25	57	55	61
Women	48	55	84	15	10	43	67	50	78	80	69
Women	41	51	77	19	19	53	70	33	64	71	68
Men	65	47	84	18	19	10	67	92	54	52	54
Women	68	59	84	20	52	53	81	58	57	63	57
Women	46	43	68	20	0	67	74	42	74	71	69
Women	48	49	60	24	57	57	89	83	74	68	69

Women	53	77	76	6	76	103	41	25	74	80	71
Women	40	45	79	21	14	67	74	67	67	80	71
Women	73	44	73	10	29	77	52	25	66	73	72
Men	68	56	70	19	0	27	56	33	62	61	61
Women	40	50	84	17	43	50	81	100	64	80	69
Men	55	58	78	20	0	13	85	58	57	52	54
Men	69	60	75	23	24	20	78	42	54	55	57
Men	75	52	79	23	0	10	78	50	54	52	54
Women	49	47	84	25	0	60	67	83	54	80	54
Men	57	49	78	21	14	63	96	75	57	64	64
Men	41	56	84	18	10	57	70	100	54	72	67
Women	51	40	84	18	14	63	78	92	57	64	64
Women	56	61	76	24	10	13	56	0	54	52	54
Women	46	50	84	24	10	33	74	25	57	61	61
Women	55	33	64	18	29	30	59	17	54	71	71
Men	68	59	80	24	5	43	81	75	54	55	54
Women	68	61	64	27	57	40	74	50	57	55	57
Women	56	55	84	26	10	27	41	42	57	52	54
Women	62	44	84	26	43	23	78	67	57	52	57
Women	39	43	78	28	0	30	93	50	66	68	57
Women	68	56	58	22	43	57	78	67	64	68	70
Men	75	85	76	23	10	27	70	42	54	61	54

Women	61	50	84	24	10	57	85	75	66	63	63
Men	68	56	79	19	33	33	81	100	67	64	67
Men	71	50	69	19	5	33	100	92	57	63	61
Women	50	54	67	18	10	37	59	33	67	66	61
Men	40	57	73	17	14	53	63	67	67	65	64
Men	71	58	71	15	43	40	78	33	62	66	61
Men	62	60	84	22	10	20	67	17	54	52	54
Women	78	46	84	29	0	3	100	75	54	52	54
Women	70	57	82	19	0	43	85	92	57	55	63
Women	50	62	70	21	33	50	96	100	57	64	61
Women	40	54	60	16	38	50	74	58	64	63	54
Women	61	50	84	22	38	60	63	75	54	52	61
Women	41	36	60	16	33	80	59	33	66	80	72
Women	44	52	79	28	0	20	78	0	57	52	54
Men	68	49	78	15	0	13	78	50	66	55	61
Women	73	54	66	22	24	37	74	75	57	61	57
Women	66	45	79	20	10	60	52	42	54	68	61
Men	51	47	82	22	0	7	93	33	54	52	54
Men	81	61	77	23	19	30	70	42	64	55	57
Women	59	56	72	24	0	37	89	92	54	59	54
Men	57	53	73	24	10	37	63	50	62	64	61
Men	73	56	74	21	33	57	85	67	54	61	54

Men	62	46	84	21	5	27	85	42	54	61	61
Men	70	61	80	24	86	83	100	100	54	63	61
Women	66	62	70	21	0	17	74	67	54	52	61
Men	46	58	81	16	14	23	81	17	64	69	64
Women	55	65	84	18	0	20	96	67	63	61	63
Men	61	62	66	18	33	50	48	50	64	64	65
Women	74	53	76	16	10	33	78	67	54	55	57
Women	44	61	76	20	5	93	59	50	54	80	72
Women	53	53	81	22	5	33	78	33	57	64	61
Women	59	48	78	23	43	83	93	75	68	68	63
Men	45	48	80	22	14	40	81	50	68	66	61
Men	41	64	84	22	14	20	70	8	54	52	54
Men	48	61	74	19	38	60	56	67	57	61	64
Women	60	52	68	23	0	53	85	92	70	71	70
Women	47	51	69	19	14	57	85	83	67	75	70
Women	56	67	70	24	29	23	85	25	54	52	54
Men	68	47	79	21	0	3	89	25	54	52	54
Women	70	56	69	16	67	27	96	33	62	52	54
Women	56	57	84	24	38	3	63	92	66	52	54
Men	68	66	71	26	10	40	85	92	54	55	54
Men	39	50	84	27	0	10	70	17	67	61	64
Men	70	66	74	15	14	13	44	17	57	52	57

Men	79	64	66	28	0	13	74	0	54	52	54
Men	58	48	69	18	14	50	67	67	57	63	64
Men	80	58	75	21	19	43	81	67	57	65	57
Women	62	52	52	23	0	23	85	75	62	59	54
Women	49	48	77	19	38	20	81	50	66	55	54
Women	64	55	75	22	48	63	85	83	76	68	71
Women	41	61	84	21	5	73	100	92	74	80	80
Women	58	52	83	22	43	57	63	50	57	63	61
Men	63	53	78	21	24	30	96	42	63	64	63
Men	58	58	73	20	38	67	85	75	61	64	61
Women	74	56	72	21	52	40	85	42	54	64	54
Women	47	58	68	18	5	20	56	42	66	65	63
Men	42	60	72	23	0	37	67	33	62	52	54
Men	66	64	60	26	38	17	93	75	62	52	54
Women	64	54	65	21	5	40	74	50	62	66	57
Women	68	49	72	22	19	27	85	50	54	59	54
Men	40	66	71	18	48	53	63	42	54	55	57
Women	43	54	75	22	62	17	89	75	54	61	61
Women	65	58	75	20	43	80	52	83	66	69	70
Women	51	47	30	18	29	43	78	58	66	72	65
Men	49	54	80	22	19	47	100	100	74	65	67
Men	67	54	84	20	0	43	89	75	54	59	54

Men	69	55	84	21	57	57	93	75	64	61	57
Women	57	60	60	18	0	3	56	0	54	52	68
Men	57	61	80	23	10	43	89	100	57	65	61
Women	43	51	84	25	0	13	81	67	55	55	54
Men	69	57	72	24	38	20	78	67	54	55	57
Men	61	58	76	10	48	70	67	17	66	63	64
Women	37	48	84	21	19	47	63	50	66	63	61
Women	45	50	84	26	0	17	70	17	62	61	63
Women	45	45	82	27	0	33	93	33	54	65	61
Women	50	58	74	23	5	43	74	50	57	64	61
Women	69	57	75	20	0	13	96	42	66	52	54
Women	72	63	63	27	29	20	59	42	66	63	67
Men	66	53	67	21	0	10	89	58	54	52	54
Men	81	55	81	30	43	3	59	8	54	52	54
Men	73	52	80	24	5	20	52	33	54	59	57
Women	46	48	80	22	14	40	81	50	68	66	61
Men	53	63	84	23	0	17	59	42	54	52	54
Women	47	55	80	21	0	30	74	83	54	65	63
Women	69	50	84	23	0	13	96	58	66	69	67
Men	54	49	76	21	29	10	78	0	54	52	54
Men	72	51	81	19	10	23	85	50	54	59	54
Women	56	66	70	25	5	43	70	75	54	61	61

Men	67	66	84	26	0	33	93	58	57	63	54
Men	66	54	79	25	52	83	74	92	57	63	57
Men	32	70	78	21	14	67	100	100	54	63	54
Men	75	60	77	21	38	20	74	50	68	64	61
Men	71	65	84	21	19	70	85	83	68	66	68
Men	55	61	82	24	19	73	67	42	54	71	63
Men	50	50	77	20	33	20	85	8	62	55	57
Men	72	48	71	20	0	20	85	17	67	52	54
Women	38	50	72	19	5	47	96	67	66	61	57
Women	50	55	72	21	38	47	78	50	64	61	63
Men	63	42	84	25	10	40	41	33	55	52	54
Men	63	57	84	21	10	80	85	83	54	61	54
Women	68	51	84	21	43	13	93	100	57	52	57
Men	57	59	80	25	24	17	93	92	54	63	54
Men	52	62	81	21	43	17	78	58	54	55	61
Men	63	56	74	17	29	40	74	67	57	63	61
Women	74	56	83	25	71	67	100	100	64	59	57
Women	57	57	82	20	0	53	74	8	64	73	64
Women	60	57	58	16	24	33	63	33	76	69	71
Men	74	67	79	27	38	40	96	75	54	52	54
Women	62	44	84	24	0	10	63	17	54	55	54
Women	66	54	84	28	33	23	89	50	67	63	61

Women	42	36	84	20	10	63	70	75	74	68	67
Women	62	52	84	22	90	50	93	83	66	63	63
Women	59	58	84	19	71	7	85	42	54	52	54
Women	75	60	68	15	38	57	70	50	76	68	61
Women	42	44	84	30	0	20	93	8	74	52	65
Women	68	57	82	19	19	33	74	75	66	61	63
Men	37	51	79	18	0	27	89	50	55	52	54
Men	61	60	84	25	19	37	63	8	66	59	54
Women	64	57	74	23	29	27	48	17	54	52	61
Men	78	58	58	15	0	27	85	33	54	55	54
Men	63	60	75	19	48	73	63	42	68	72	68
Men	77	59	81	20	14	50	93	67	57	64	57
Men	56	47	84	25	10	30	48	8	61	61	61
Women	73	56	63	19	48	40	74	58	61	52	61
Men	73	46	83	29	0	7	81	17	57	52	54
Men	59	57	70	24	14	17	30	0	54	52	54
Men	62	48	77	21	5	43	81	50	66	71	68
Men	61	60	76	22	0	20	70	33	57	63	57
Men	67	62	74	20	38	87	89	92	57	74	71
Men	55	46	80	19	0	30	81	42	54	52	54
Women	48	52	84	22	38	47	78	67	63	66	63
Women	57	52	80	28	24	67	74	75	64	61	61

Women	46	48	84	24	0	20	70	42	57	59	57
Men	74	59	84	25	14	10	93	8	54	52	54
Women	59	55	84	21	10	30	52	33	54	64	54
Women	53	52	80	22	33	27	81	83	68	64	61
Women	55	54	84	22	5	37	78	50	63	59	63
Women	38	46	84	28	0	30	96	83	57	59	57
Men	51	57	77	18	24	40	67	50	57	66	61
Men	66	70	74	18	33	80	67	58	62	68	61
Men	70	51	77	20	5	10	100	58	54	55	57
Men	53	59	63	18	10	17	67	25	62	55	61
Women	71	57	70	18	14	47	70	58	54	63	61
Women	59	61	78	15	24	77	70	67	54	71	67
Men	56	69	75	26	0	60	81	25	62	80	65
Women	72	66	76	17	57	60	96	67	54	61	63
Women	79	56	84	22	24	20	56	42	57	55	61
Men	67	62	68	23	19	23	81	75	62	52	61
Women	60	54	84	9	0	83	52	58	54	68	61
Women	54	32	84	30	0	17	89	25	57	61	57
Men	58	23	84	28	0	3	85	33	54	55	54
Men	75	67	53	15	19	67	15	42	62	80	76
Men	72	40	84	23	0	3	81	0	64	52	54
Men	71	61	82	22	0	10	100	58	54	52	54

Women	79	37	84	27	43	17	81	42	62	63	54
Men	71	64	84	22	33	40	85	75	55	52	54
Women	77	59	81	29	0	20	100	42	54	52	54
Men	42	58	84	22	0	37	78	67	57	61	57
Men	71	67	76	23	29	30	74	92	55	52	54
Men	78	51	75	21	43	30	93	67	54	52	54
Women	47	65	80	20	10	77	78	33	57	80	70
Men	40	49	83	22	14	23	93	33	64	64	63
Women	68	61	81	17	29	80	93	92	57	71	63
Women	53	63	70	16	38	60	44	33	78	75	73
Women	60	55	73	20	24	20	70	17	57	59	57
Men	63	58	84	19	0	3	56	0	55	55	54
Men	60	54	84	25	14	20	96	92	55	52	54
Women	43	49	84	26	0	7	100	58	66	68	68
Women	69	48	84	23	0	30	74	42	55	52	54
Women	48	54	83	18	57	53	56	42	78	72	71
Women	55	49	83	17	14	13	100	50	55	55	54
Women	58	54	77	24	33	43	78	67	68	69	68
Men	50	52	83	21	0	7	74	8	54	52	54
Men	39	66	62	22	10	40	63	17	57	66	63
Men	64	71	73	22	14	37	96	75	57	52	54
Men	51	65	79	22	14	17	67	17	54	55	57

Men	64	60	48	18	0	3	7	0	55	52	54
Women	42	51	72	21	5	43	74	33	62	71	64
Men	55	62	84	20	43	37	67	33	54	52	54
Women	60	53	78	22	38	20	67	0	54	59	57
Women	45	63	84	16	67	100	19	67	80	80	80
Men	77	55	80	24	29	50	67	58	54	52	54
Men	71	59	68	18	10	50	30	33	54	80	69
Women	63	46	79	22	29	73	67	50	68	71	68
Women	82	58	60	19	29	57	63	67	64	72	61
Men	61	67	80	22	14	23	96	17	62	63	61
Men	37	41	74	28	10	77	81	75	67	73	65
Men	71	50	72	27	0	23	81	33	57	52	54
Women	50	62	80	21	0	37	78	75	57	52	54
Men	65	64	84	18	14	67	81	42	57	73	64
Women	71	56	74	21	43	47	81	92	55	63	54
Men	65	59	80	26	0	20	93	67	62	61	54
Women	74	53	81	25	10	20	89	75	57	55	54
Women	66	71	71	22	10	27	52	33	66	71	70
Women	69	69	71	23	14	10	41	8	54	61	54
Women	56	59	76	19	43	30	93	92	54	55	54
Men	35	59	78	19	0	13	74	0	57	52	61
Women	57	64	70	9	43	90	56	17	67	80	67

Men	70	60	82	22	29	47	78	83	54	52	54
Men	65	61	71	20	0	27	63	33	64	52	54
Men	52	43	76	25	0	13	70	25	54	59	54
Women	75	52	73	21	5	30	74	67	57	61	57
Men	61	47	84	21	0	30	67	50	65	63	65
Women	26	41	76	22	0	23	78	33	66	61	65
Men	72	61	84	15	0	43	81	67	54	63	57
Women	58	53	72	28	0	17	85	17	66	65	57
Women	76	40	81	29	0	7	78	8	62	63	54
Men	79	55	74	27	19	27	59	50	54	55	57
Men	43	59	73	19	29	60	74	42	63	72	63
Men	69	41	64	21	29	20	89	75	54	61	54
Men	64	61	67	19	29	3	67	0	54	52	54
Women	68	55	72	19	38	40	74	50	61	64	61
Women	77	58	72	25	0	3	100	92	54	52	54
Women	68	57	76	18	43	7	78	75	57	52	57
Women	45	67	65	24	14	30	81	67	57	55	63
Women	42	32	84	26	0	83	52	58	64	63	61
Women	69	54	84	22	38	50	93	83	67	61	63
Women	51	70	81	26	14	43	52	33	61	72	61
Women	47	57	84	17	14	60	100	25	54	68	54
Women	53	55	84	19	29	37	81	17	57	65	57

Men	62	57	71	21	52	13	85	58	57	52	54
Women	63	60	68	18	0	27	78	42	55	59	54
Women	50	47	84	23	0	17	81	25	57	59	57
Women	46	44	84	24	5	67	100	75	80	59	63
Men	66	51	80	22	67	27	74	42	57	52	54
Women	39	52	84	17	24	50	78	33	74	66	61
Men	67	63	83	22	0	33	81	92	54	52	54
Women	65	50	72	20	19	43	74	42	64	71	64
Women	73	57	56	24	0	30	81	17	61	55	61
Men	74	60	27	19	10	13	70	50	66	55	57
Men	69	59	75	17	52	70	78	50	66	65	61
Women	70	52	53	20	14	13	63	67	54	52	54
Women	74	51	77	25	71	37	96	100	55	63	54
Men	70	58	72	21	67	23	78	50	64	55	54
Men	64	66	66	19	57	50	89	75	63	52	63
Women	57	62	66	24	0	33	93	83	68	73	69
Women	57	60	82	17	5	27	96	50	64	59	57
Women	66	56	79	20	14	43	89	50	54	59	57
Men	66	73	74	19	67	37	78	50	54	52	54
Women	51	48	82	21	10	57	93	58	76	72	61
Men	68	60	71	19	5	43	100	83	61	61	61
Women	50	48	71	20	10	27	89	42	57	59	57

Women	76	63	72	14	29	27	63	50	80	72	71
Men	35	56	81	18	14	67	89	58	57	80	70
Women	74	59	66	14	14	37	59	25	66	64	63
Women	56	36	84	21	0	27	74	92	57	68	61
Men	80	76	74	25	14	50	74	42	64	61	61
Men	73	63	79	22	0	7	74	42	66	55	57
Men	67	43	84	30	0	33	89	8	62	59	64
Women	53	56	76	25	5	30	63	8	61	65	61
Men	61	60	48	18	0	33	81	75	78	72	71
Women	56	51	84	20	24	40	85	50	66	69	61
Men	64	74	66	16	14	60	48	58	66	73	70
Women	39	49	81	21	14	43	74	17	64	71	64
Women	74	64	52	17	5	33	81	75	64	72	70
Women	46	56	69	20	52	80	78	50	64	66	64
Men	75	69	80	17	5	23	85	42	54	71	61
Men	63	45	80	24	43	17	96	67	54	52	54
Women	54	62	70	21	19	57	63	33	64	64	63
Women	77	58	84	6	29	57	74	42	54	63	61
Men	79	64	82	18	0	33	96	58	54	52	54
Men	73	49	80	20	0	27	96	67	55	52	54
Women	77	54	73	17	29	70	74	42	64	64	68
Men	81	62	64	19	10	30	74	58	54	52	54

Women	79	60	77	21	38	10	74	42	55	52	54
Men	81	63	75	19	52	73	67	58	76	64	64
Women	81	59	67	17	0	20	63	67	62	59	54
Women	45	54	69	26	43	40	78	58	63	65	63
Women	39	51	75	20	0	23	70	17	62	52	54
Women	44	43	58	14	10	70	70	75	80	71	70
Men	61	52	68	23	0	30	74	33	80	72	71
Women	60	50	81	19	10	40	89	58	55	52	54
Women	68	53	82	21	29	93	93	83	54	80	71
Women	44	36	84	28	5	30	93	17	68	59	57
Men	48	72	80	15	5	33	81	83	54	61	63
Women	63	58	69	19	33	37	41	33	68	52	61
Women	52	48	81	20	33	83	78	67	68	71	67
Women	48	61	84	19	24	70	70	67	70	71	70
Men	63	53	75	21	24	23	89	8	62	59	54
Men	62	58	80	24	14	33	59	25	63	59	63
Men	50	60	84	24	0	20	67	8	67	66	67
Men	56	57	71	22	0	33	85	50	66	71	57
Men	75	55	82	23	14	43	89	100	68	52	54
Women	68	50	79	20	0	50	52	8	68	73	68
Women	70	66	76	20	38	47	78	25	64	63	64
Women	45	56	84	26	0	27	100	83	57	66	57

Women	66	65	72	23	0	43	89	42	54	55	57
Women	66	65	72	23	0	43	89	42	54	55	57
Women	66	65	72	23	0	43	89	42	54	55	57
Women	66	65	72	23	0	43	89	42	54	55	57
Women	66	65	72	23	0	43	89	42	54	55	57
Women	66	65	72	23	0	43	89	42	54	55	57
Women	75	53	77	27	5	13	74	58	54	52	61
Men	64	61	84	22	14	13	52	0	54	59	54
Women	73	61	74	18	19	47	74	67	78	73	74
Women	54	52	57	24	24	40	96	100	68	72	70
Women	50	57	82	27	0	13	70	67	54	59	54
Men	55	67	63	24	0	33	78	42	54	59	61
Women	51	69	78	18	0	47	93	100	66	69	63
Women	69	57	84	24	5	50	74	42	64	74	63
Men	64	62	66	19	29	10	67	0	54	55	54
Men	70	63	30	21	24	23	74	67	55	61	54
Women	54	53	76	20	5	17	85	25	54	52	54
Men	64	72	66	21	5	67	85	83	57	68	57
Men	62	65	84	25	0	13	70	50	62	52	54
Men	59	51	80	22	5	73	59	42	54	71	65
Women	56	60	75	20	48	40	63	50	54	59	54
Women	46	50	84	18	29	27	85	75	64	63	61

Men	64	49	76	19	0	37	89	100	54	55	57
Women	48	50	77	10	0	47	89	100	64	63	64
Women	66	48	67	16	38	10	78	75	62	52	54
Women	55	54	84	23	0	17	85	25	61	59	61
Women	50	53	77	17	10	23	52	42	57	61	61
Women	46	64	79	19	29	73	78	75	67	80	73
Women	60	47	80	17	19	33	41	33	68	65	67
Women	66	51	79	19	24	37	67	92	57	63	61
Women	43	58	57	17	33	67	74	67	62	71	71
Women	68	54	84	29	14	13	96	42	57	52	54
Women	58	62	61	24	10	73	81	42	64	64	63
Women	53	54	76	20	29	63	74	50	76	68	64
Women	64	59	81	20	0	23	85	33	66	52	54
Women	46	56	82	24	0	40	81	92	54	59	57
Men	35	58	60	27	0	7	89	50	66	52	54
Men	26	55	76	17	0	20	48	8	54	52	54
Men	64	50	80	21	5	27	85	58	54	64	57
Women	57	53	83	25	38	17	81	8	54	59	54
Women	40	58	76	20	5	37	85	67	80	74	70
Women	64	51	82	26	14	40	100	58	54	55	54
Women	55	40	81	20	14	90	59	17	80	80	70
Women	69	55	78	27	0	20	100	58	57	55	57

Women	59	36	81	30	5	30	93	25	67	68	68
Women	57	80	54	14	76	93	41	75	80	80	80
Men	65	60	81	21	38	50	70	92	66	55	61
Women	42	58	76	20	5	33	67	67	61	63	61
Women	64	69	60	20	0	17	74	42	55	59	54
Women	63	54	80	23	0	33	96	50	65	65	65
Men	71	66	76	25	0	3	93	25	57	52	57
Women	70	60	69	23	5	67	96	75	66	65	61
Men	67	60	74	23	19	47	89	42	64	63	61
Women	67	59	70	24	14	37	81	67	74	52	61
Women	44	58	78	20	57	33	81	42	68	65	65
Men	60	55	74	21	29	50	74	33	62	64	61
Women	57	57	80	24	0	50	81	100	62	61	67
Women	65	65	76	22	33	67	96	100	76	63	54
Women	60	49	81	27	0	13	85	58	62	55	61
Men	64	57	80	20	10	33	81	33	54	52	54
Women	57	56	69	26	5	23	70	33	54	63	61
Women	58	51	70	22	19	17	56	17	54	52	54
Men	51	65	76	18	5	33	85	83	54	61	61
Women	65	76	67	25	29	57	26	67	57	74	74
Men	69	61	76	19	29	63	59	67	57	64	57
Women	53	44	84	30	0	3	44	0	54	52	54

Women	53	50	81	21	5	17	89	100	54	52	54
Women	64	39	78	30	0	7	96	25	57	55	54
Women	52	62	59	22	62	77	85	75	57	66	64
Women	52	62	59	22	62	77	85	75	57	66	64
Women	52	62	59	22	62	77	85	75	57	66	64
Women	52	62	59	22	62	77	85	75	57	66	64
Women	52	62	59	22	62	77	85	75	57	66	64
Men	35	54	81	24	0	20	59	58	62	61	61
Women	63	57	81	18	38	87	78	92	64	80	68
Men	72	61	75	18	0	40	81	92	54	65	61
Women	69	60	78	22	43	47	85	83	62	69	65
Women	56	39	81	26	0	43	85	58	64	65	61
Women	57	50	74	21	33	43	89	83	54	63	54
Women	35	57	79	23	0	30	74	42	61	63	61
Women	48	55	81	22	71	47	96	42	67	65	61
Women	62	51	82	24	33	7	93	92	64	52	57
Women	38	48	74	24	29	20	78	83	57	64	57
Women	33	58	79	11	38	97	41	17	67	72	67
Women	33	36	84	18	52	87	70	75	66	73	73
Women	68	59	79	19	5	57	89	75	66	66	57
Men	58	69	56	16	43	67	89	83	67	71	64
Women	46	58	80	22	0	20	81	17	66	61	57

Women	49	56	71	19	57	20	89	83	66	66	61
Women	68	66	75	24	14	17	74	67	66	68	61
Women	69	70	53	15	19	77	85	100	57	73	68
Men	41	64	78	20	0	30	78	50	64	55	61
Women	72	63	77	22	0	83	93	100	54	72	63
Men	51	65	72	25	57	23	59	0	55	64	54
Women	77	48	79	26	48	30	93	8	57	52	57
Men	67	66	64	19	24	27	67	75	54	52	54
Women	65	55	72	19	29	3	63	25	57	55	54
Women	73	58	70	17	48	70	52	50	68	71	74
Women	53	46	82	27	38	60	100	75	54	66	61
Women	49	34	70	19	5	30	70	67	62	68	70
Women	47	57	83	21	43	47	81	83	62	61	54
Women	45	63	84	24	62	73	70	67	76	72	65
Women	52	49	80	14	48	80	70	75	55	52	54
Women	57	54	84	27	29	67	67	58	78	64	63
Women	73	45	84	24	0	13	100	42	57	59	54
Women	59	52	82	23	0	7	89	42	62	52	54
Men	67	60	64	25	5	53	74	75	57	52	57
Women	83	62	71	19	14	27	85	67	62	55	54
Men	47	59	62	18	24	53	67	58	66	65	64
Men	46	41	81	26	5	47	78	50	64	71	65

Women	75	61	79	19	33	47	70	67	66	69	65
Women	68	51	79	17	19	60	63	42	54	52	54
Men	65	53	79	20	33	77	74	75	54	59	65
Men	76	60	62	21	14	23	78	58	54	52	54
Men	59	56	83	23	67	27	93	83	54	52	57
Women	40	63	84	21	29	20	93	33	55	52	54
Women	67	46	84	26	0	63	100	83	54	64	63
Women	68	54	82	18	38	57	74	58	67	69	63
Men	47	61	84	18	48	57	74	100	54	68	61
Women	57	50	84	19	0	17	85	83	57	52	54
Women	44	51	84	20	43	20	100	100	62	61	54
Women	69	58	77	17	29	37	78	58	57	61	57
Women	75	59	78	25	71	53	96	92	64	61	67
Men	25	60	83	23	0	30	67	33	62	61	61
Women	76	60	73	26	0	27	85	50	62	55	54
Men	24	45	78	20	0	20	85	33	76	71	64
Men	77	60	59	19	43	43	59	58	54	52	54
Women	81	55	66	20	43	53	70	58	61	63	61
Men	72	53	72	23	38	17	56	42	67	55	54
Women	61	55	82	22	48	37	89	100	54	52	54
Men	81	61	84	18	48	40	74	42	62	68	57
Men	61	53	75	19	38	50	74	67	68	64	68

Men	62	66	84	19	14	27	41	50	61	63	61
Men	76	54	84	15	38	57	74	50	62	63	57
Women	73	58	84	19	29	40	63	58	66	55	57
Men	65	51	84	20	10	13	93	33	62	59	54
Women	81	57	78	17	62	70	89	75	54	64	54
Women	59	53	77	18	24	43	93	83	67	68	63
Women	46	54	80	18	29	60	78	100	57	61	57
Men	57	63	80	25	0	60	89	58	66	65	57
Women	45	58	78	18	0	7	85	58	57	52	54
Women	51	55	66	18	52	53	81	67	58	59	57
Women	62	60	30	15	0	67	78	67	57	63	63
Women	45	41	84	20	0	17	67	25	57	61	61
Women	33	64	52	14	43	83	56	42	64	71	70
Women	56	62	73	17	24	97	74	33	54	80	74
Women	72	61	84	18	52	27	89	83	54	63	61
Women	44	50	84	25	0	27	74	33	74	65	61
Women	69	63	81	16	48	53	70	67	62	66	61
Women	54	49	78	26	0	13	44	0	61	55	61
Women	66	52	83	19	0	10	59	8	54	52	54
Men	76	67	52	19	0	47	63	33	54	68	65
Women	70	61	79	22	43	37	85	75	64	55	61
Men	64	53	79	21	5	20	70	17	65	59	65

Women	58	63	84	21	5	60	78	92	61	66	61
Women	45	54	30	15	43	47	93	67	66	80	67
Men	61	75	58	24	14	10	85	67	55	52	54
Women	67	56	79	22	5	20	100	50	55	59	54
Women	40	60	84	22	0	27	85	58	57	66	61
Women	50	60	84	25	0	60	67	83	54	80	54
Men	61	60	63	19	5	10	63	42	54	55	54
Men	68	60	83	18	14	20	96	100	54	59	54
Women	59	60	72	19	5	33	59	50	61	61	61
Women	51	60	77	27	0	10	96	42	54	55	54
Women	47	60	77	15	0	23	41	42	74	69	54
Women	46	60	84	27	24	7	100	83	54	55	54
Men	78	60	66	18	0	7	67	8	54	52	54
Women	59	60	83	27	0	20	44	0	54	52	54
Women	51	60	71	15	5	17	67	25	54	55	54
Women	49	60	59	14	24	80	56	67	74	80	74
Women	59	60	76	25	14	83	67	42	67	69	63
Men	74	60	76	20	48	33	78	67	54	55	61
Men	63	60	84	17	38	73	78	67	78	71	71
Women	47	60	83	28	0	23	74	25	54	59	57
Men	70	60	83	22	0	27	89	67	66	61	61
Men	80	60	52	20	0	30	33	25	62	55	65

Women	67	60	84	29	0	13	81	17	54	52	61
Men	64	60	73	23	29	57	81	42	63	52	63
Men	26	60	58	19	33	47	26	0	66	68	70
Women	71	60	84	24	14	20	78	33	57	52	54
Women	47	60	68	20	0	67	74	42	74	71	69
Women	72	60	56	13	62	83	70	75	76	80	76
Men	59	60	70	20	29	20	59	58	69	68	69
Women	48	60	63	22	24	37	59	33	74	66	65
Women	73	60	77	21	67	47	78	100	64	63	64
Women	72	60	65	18	10	40	81	83	66	63	69
Women	41	59	76	23	14	10	89	42	62	52	61
Women	50	59	75	25	5	63	81	67	62	61	61
Women	55	59	84	27	19	87	63	50	66	80	72
Women	48	59	79	18	0	33	78	75	57	68	61
Women	41	59	59	14	19	70	56	67	57	71	73
Women	44	59	73	17	0	40	85	50	61	61	61
Women	52	59	83	21	0	30	78	50	66	59	54
Women	50	59	72	18	14	30	33	17	66	71	71
Men	74	59	74	20	10	17	67	42	62	55	54
Women	63	59	80	25	19	23	85	83	57	64	61
Men	62	59	70	16	86	50	100	67	54	55	61
Men	37	59	75	14	24	47	41	42	66	66	67

Women	77	59	69	19	29	63	70	58	57	61	63
Women	70	59	68	13	29	87	59	58	68	80	70
Men	67	59	80	21	48	53	74	75	54	52	54
Men	60	59	68	18	10	20	37	17	57	52	57
Men	57	58	79	23	29	27	78	58	78	59	63
Men	76	58	56	17	43	83	89	83	62	63	54

Abbreviations. LSS, Liverpool Stoicism Scale; MSPSS, Multidimensional Scale of Perceived Social Support; LOT-R, Life Orientation Test-Revised; MAC, Mini-Mental Adjustment to Cancer; MAC_HH: Helplesness. MAC_ ÁP: Anxious preoccupation; MAC_PA: Positive Attitude; MAC_CA: Cognitive Avoidance; BSI, Brief Symptom Inventory; BSI_Som: Somatization; BSIAn: Anxiety; BSIDe: Depression
