# Supplementary material for: Stoic attitude in patients with cancer from the NEOcoping study: Cross-sectional study
Source: PLoS One. 2022 Jul 28;17(7):e0269712. doi: 10.1371/journal.pone.0269712 (PMC9333303; doi:10.1371/journal.pone.0269712)
Supplement: Attachment — Submitted filename: PONE-D-20-38896_R1 (1).pdf [file pone.0269712.s003.pdf]

PLOS ONE

Stoic attitude in patients with cancer from the NEOcoping study: cross-sectional study

--Manuscript Draft--

Manuscript Number:	PONE-D-20-38896R1
Article Type:	Research Article
Full Title:	Stoic attitude in patients with cancer from the NEOcoping study: cross-sectional study
Short Title:	Stoic attitude in patients with cancer
Corresponding Author:	Caterina Calderón<br>University of Barcelona<br>Barcelona, Barcelona SPAIN
Keywords:	Brief Symptom Inventory; Liverpool Stoicism Scale; Mental Adjustment to Cancer; Multidimensional scale of perceived social support; Revised Life Orientation Test; Stoicism
Abstract:	Aim: The aim was to explore stoicism in patients with cancer, and to assess its correlation with coping, psychological distress, optimism, perceived social support, and estimated risk of recurrence and toxicity.<br>Method: NEOcoping is a multicenter, cross-sectional study. Participants' data were collected using a standardized, self-report form and LSS, MSPSS, Mini-MAC, BSI-18, and LOT-R questionnaires. Linear regression analyses were used to assess the association between stoicism and distress scores in both genders. A total of 932 individuals with non-metastatic, resected cancer were recruited.<br>Results: Males perceived a higher risk of recurrence and toxicity with adjuvant chemotherapy and obtained higher stoic attitude scores than females. Women scored higher on somatization, depression, and anxiety. Patients with high stoicism scores were older and experienced more maladaptive coping (helplessness, anxious preoccupation), and depression, while those with lower stoicism scores had greater perceived social support, positive attitude, and optimism. In both males and females, stoicism correlated negatively with perceived social support, positive attitude, and optimism and positively with helplessness, anxious preoccupation, and depression. In men, stoicism also correlated positively with a perception of a higher risk of recurrence and with anxiety, and in women, with age. Of the variance in stoicism, 15.6% was accounted by perceived social support, optimism, and anxious preoccupation in men and 16.9% was explained by perceived social support, optimism and age in women.<br>Discussion: Stoic attitude was associated with worse coping, greater psychological distress, decreased optimism, and perception of less social support in this series of patients with cancer.<br>Aim: The aim was to explore stoicism in patients with cancer, and to assess its correlation with coping, psychological distress, optimism, perceived social support, and estimated risk of recurrence and toxicity.<br>Method: NEOcoping is a multicenter, cross-sectional study. Participants' data were collected using a standardized, self-report form and LSS, MSPSS, Mini-MAC, BSI-18, and LOT-R questionnaires. Linear regression analyses were used to assess the association between stoicism and distress scores in both genders. A total of 932 individuals with non-metastatic, resected cancer were recruited.<br>Results: Males perceived a higher risk of recurrence and toxicity with adjuvant chemotherapy and obtained higher stoic attitude scores than females. Women scored higher on somatization, depression, and anxiety. Patients with high stoicism scores were older and experienced more maladaptive coping (helplessness, anxious preoccupation), and depression, while those with lower stoicism scores had greater perceived social support, positive attitude, and optimism. In both males and females, stoicism correlated negatively with perceived social support, positive attitude, and optimism and positively with helplessness, anxious preoccupation, and depression. In men, stoicism also correlated positively with a perception of a higher risk of recurrence and with anxiety, and in women, with age. Of the variance in stoicism, 15.6% was accounted by perceived social support, optimism, and anxious preoccupation in men and 16.9% was explained by perceived social support, optimism and age in women.<br>Discussion: Stoic attitude was associated with worse coping, greater psychological distress, decreased optimism, and perception of less social support in this series of

Powered by Editorial Manager® and ProduXion Manager® from Aries Systems Corporation

	patients with cancer.
**Order of Authors:**	David Gomez
	Alberto Carmona-Bayonas
	Raquel Hernandez
	Oliver Higuera
	Jacobo Rogado-Revuelta
	Vilma Pacheco-Barcia
	María Valero
	Mireia Gil Raga
	Mª Mar Muñoz
	Rafael Carrión-Galindo
	Paula Jimenez-Fonseca
	Caterina Calderón
**Response to Reviewers:**	Reviewer #1: This manuscript explored the association of stoicism with coping, psychological distress, optimism, perceived social support, and estimated risk of recurrence in cancer patients. The present study has several strengths: a well-written manuscript, clinical relevance of the study objective to the cancer survivorship and a relatively large sample size. Nevertheless, the reviewer has the following comments and criticism on the conceptual framework and methodology of the study, particularly on the incremental value of stoicism over existing cancer coping strategies.<br>Well aware of the value your critical reading brings to our text, we would like to begin by thanking the reviewer for having read the text and for considering that it may be of scientific interest and for having captured the highlights and strengths of this work. Please find below our response to each of the comments made by the reviewer.<br>1) Introduction: Stoicism is a interesting concept that has rarely been examined in the context of coping with cancer. The authors brought up this innovative construct and could potentially contribute to the existing literature. However, the current introduction is too brief and much more information is needed to establish the importance of the concept of stoicism in the context of cancer. The first two paragraphs are just overall introduction of stoicism without specific relevance to cancer. The authors need to provide a more focused background of the study while adding more citations from relevant literature. There are currently only 6 references in the Introduction!<br>In response to this question, we have expanded the explanation of the relevance and implication of stoicism in cancer patients and the available scientific evidence on this issue.<br>To clarify this point, the following paragraph has been added to the Introduction section:<br>“A study of men with prostate cancer of any stage suggests that stoicism is neither adaptive nor maladaptive for distress and quality of life outcomes and is not highly correlated with male self-esteem or psychological flexibility. The authors hypothesized that this may mean that men use stoicism to cope with prostate cancer in line with their own masculine values*. In another study by Mah et al., younger, and older patients with advanced cancer showed unique biopsychosocial correlates of pain-related stoicism and caution** Cognitive avoidance of distressing thoughts was intrapersonal, associated with greater concealment, self-doubt, and reticence in the elderly. Older adults were more likely to regulate emotions, to be less expressive about negative events and avoidance of negative information in relation to a more stoic attitude.”<br>*McAteer G, Gillanders D. Investigating the role of psychological flexibility, masculine self-esteem and stoicism as predictors of psychological distress and quality of life in men living with prostate cancer. Eur J Cancer Care (Engl). 2019;28(4):e13097..<br>**Mah K, Tran KT, Gauthier LR, et. Do Correlates of Pain-Related Stoicism and Cautiousness Differ in Younger and Older People With Advanced Cancer? J Pain. 2018;19(3):301-316.<br>2) What does the last sentence in Introduction “Finally, we have developed a model of stoicism correlating with coping, psychological stress, optimism, and perceived social support” mean? Is it one of the study objectives?

Powered by Editorial Manager® and ProduXion Manager® from Aries Systems Corporation

The final sentence of the introduction does not correspond to either of the two objectives mentioned above and has therefore been deleted.
3) Given the cross-sectional nature of this study, implications of the current results are limited and measurement of the estimated risk of future recurrence might not be appropriate and relevant.
As the reviewer refers, cross-sectional nature of this study is a limiting factor and this is stated in the first limitation in the discussion section: "First, given its cross-sectional design, a causal interpretation cannot be made of the association between stoicism and psychological variables. Stoic perspective could only be assessed at a given point in time and not throughout the entire process of adapting to cancer."
Patients were asked about their perceived risk of recurrence before starting chemotherapy adjuvant/complementary to surgery with curative intent. Since tumor stage was similar, the influence of stoicism, gender and other psychological variables on patients' perception of their risk of cancer recurrence was assessed.
Therefore, the patient's perception of their risk of cancer recurrence was considered, but no calculation of the risk of recurrence was made based on any of the variables studied. Therefore, the analyses do not establish causal relationships in oncological outcomes.

4) Mini-MAC: In page 4, the authors wrote that the Mini-MAC assesses five cognitive coping responses, but they only listed helplessness, anxious preoccupation, positive attitude, and cognitive avoidance as the 4 factors. It seems the authors have somehow adopted the 4-factor structure for Mini-MAC advocated in the Norwegian version they cited in [10]. However, as the authors have quoted, the Mini-MAC is generally perceived to have 5 factors with “positive attitude” being distinguished into fighting spirit (4 items) and 5) fatalism (5 items). The authors need to better a stronger justification not to opt for the conventional factor structure for the Mini-MAC given it is a well-established and validated scale of cancer coping.
The authors of this paper validated the Spanish version of the Mini-MAC in a Spanish series of cancer patients*.
The results of the factor analysis indicated a 4-factor structure. Three subscales have similar psychometric properties to the Helplessness, Anxious preoccupation, and Cognitive avoidance subscales of the original Mini-MAC and the Fighting Spirit and Fatalism subscales were combined into the Positive Attitude scale. The scale scores derived from the four factors showed acceptable accuracy for individual measures, as well as stability across test-retest assessments at 6 months. Validity assessments found meaningful relations between the derived scale scores, and Brief Symptom Inventory (BSI) depression and anxiety scores and Functional Assessment of Chronic Illness Therapy (FACIT) spiritual well-being scores.
Therefore, given that our team had validated and confirmed in previous work the accuracy of a 4-factor based scale, it was decided to use it in this work.
In the method section, we have added the following clarification regarding this point: “The 29-item mini-MAC assesses five cognitive coping responses: helplessness, anxious preoccupation, fighting spirit, fatalism and cognitive avoidance. For this study, the version with a 4-factor structure validated in Spanish was used, grouping fighting spirit and fatalism into a single factor, positive attitude*.”
*Calderon C, Lorenzo-Seva U, Ferrando PJ, et al. Psychometric properties of Spanish version of the Mini-Mental Adjustment to Cancer Scale. Int J Clin Health Psychol. 2021;21(1):100185. doi: 10.1016/j.ijchp.2020.06.001.
5) Incremental value of stoicism: Following the last comment on Mini-MAC, the authors need to delineate the conceptual distinction between stoicism and existing cancer coping strategies such as fatalism and cognitive avoidance as Mini-MAC factors. It seems to the reviewer that stoicism has conceptual overlap with both fatalism and cognitive avoidance. It is essential to specify the conceptual difference between stoicism and existing cancer coping strategies to establish the need for the present study.
The term fatalism refers to the belief in the determinism of events, directed by causes independent of human will. This notion of fatalism carries a negative connotation. While Stoicism is based on the notion of control over life-disturbing emotions, the goal is based on tolerance and self-control. We have made changes to the introduction to delineate the concepts.
6) Analysis: The authors conduct tests on gender and age differences in stoicism and other psychological variables. Did they consider ANOVAs to test the potential interaction effects between age and gender? This should be feasible given the large

Powered by Editorial Manager® and ProduXion Manager® from Aries Systems Corporation

sample size. The findings presented in Tables 2 – 4 are mostly t-tests and correlational analysis, which are rather basic and do not take full account of the complex relationships among the variables.
You are right, it would be interesting to analyze the interaction between stoicism and sex and age and we will take that into account for future studies. The objective of the present study was to analyze the differences between patients with high and low levels of stoicism.
7) Why did the authors dichotomize the continuous stoicism score into high and low stoicism subgroups? What is the added value of conducting t-tests across the high-low subgroups over correlation as in Table 4? The results in Tables 3 and 4 appear to be redundant without new information added.
To carry out the statistical analyzes presented in tables 2 and 4, we rely on the scientific literature that indicates that significant differences are observed between men and women in stoicism. The correlation of stoicism with the psychological variables according to sex was carried out first, in order to know which of these would be predictive in explaining stoicism (Table 5), also differentiating it by sex.
8) The authors conducted the correlation analyses in male and female subsamples separately in Table 4. Are the gender differences significant? Most of the correlations in Table 3 are rather small and < 0.30. Do they bear much clinical relevance to the participants?
To carry out the statistical analyzes presented in tables 2 and 4, we rely on the scientific literature that indicates that significant differences are observed between men and women in stoicism. The correlation of stoicism with the psychological variables according to sex was carried out first, in order to know which of these would be predictive in explaining stoicism (Table 5), also differentiating it by sex.

9) Most of the variables in the study are coping strategies (social support, Mini-MAC, optimism). Why did the authors use these variables to predict stoicism in Table 5? Is stoicism perceived as a predictor of cancer symptoms/distress or outcome of other coping strategies in this study? Even though this study is only cross-sectional in nature and could not answer the causal direction among the study variables, it would be better if the authors could provide a clearer conceptual framework of the study variables related to the main construct of stoicism.
Our study aims to explain the percentage of explained variance of various psychological variables in stoicism, it does not try to explain how stoicism develops, for this we would need to carry out a longitudinal study. Following your recommendations, we have improved the conceptual framework of the introduction.
10) The authors wrote they conducted structural equation models for the regression analyses. However, a lot of information is missing for the SEM analysis, such as model specification, model fit, model modification, control variables in the model. How can they conduct SEM in SPSS 23.0 and have F-values in the results in SEM analysis? Presentation of the last part of results is too brief for the reviewer and readers to understand. Did the authors use latent factors of the predictors or outcomes in the SEM analysis?
As described in Data Analysis, in the manuscript we use two linear regression analyzes to predict the association between stoicism and psychological scales in men and women. In subsequent statistical analyses, stoicism served as the criterion variable in regression analyses, while social support (according to the MSPSS), coping strategies (Mini-MAC), psychological distress (BSI-18) and optimism (LOT-R) were used as predictors.
We have not used SEM. We are sorry.

Reviewer #2: In the article there is no theoretical background of concepts measured in the study, except stoic attitude. The other variables included in the research should be also described in the introduction.
In response to the reviewer's request, we have added the following paragraph in the introduction section relating to the other items, coping, psychological distress, optimism, and perception of social support:
“As cancer is a stressful event associated with fear and prognostic uncertainty, it is common for patients to employ coping strategies to deal with it. Stoicism and emotional distress (anxiety, depression, and somatization) may interfere with coping by increasing anxious preoccupation*. Dispositional optimism may act as a resource that maintains a positive mood, provides a more flexible coping style to face with the

Powered by Editorial Manager® and ProduXion Manager® from Aries Systems Corporation

	inability to control stressful stimuli, and protects patients from the possible negative effects of cancer treatment and cancer itself in case of recurrence**,***. Likewise, social support, defined as the perception that others are willing to help, appears to operate similarly to optimism and is a critical factor in decreasing negative psychological affect in individuals with cancer****."<br>*Jimenez-Fonseca P, Calderón C, Hernández R, et al. Factors associated with anxiety and depression in cancer patients prior to initiating adjuvant therapy. Clin Transl Oncol. 2018;20(11):1408-1415. doi: 10.1007/s12094-018-1873-9.<br>**Ciria-Suarez L, Calderon C, Fernández Montes A, et al. Optimism and social support as contributing factors to spirituality in Cancer patients. Support Care Cancer. 2021;29(6):3367-3373. doi: 10.1007/s00520-020-05954-4.<br>***Ciria-Suarez L, Jiménez-Fonseca P, Palacín-Lois M, et al. Breast cancer patient experiences through a journey map: A qualitative study. PLoS One. 2021 Sep 22;16(9):e0257680. doi: 10.1371/journal.pone.0257680.<br>****Calderon C, Gomez D, Carmona-Bayonas A, et al. Social support, coping strategies and sociodemographic factors in women with breast cancer. Clin Transl Oncol. 2021;23(9):1955-1960. doi: 10.1007/s12094-021-02592-y<br>In the methodological part, we have little information about specific cancer diagnosis. Cancer patients have different e. g. coping styles or depressive symptoms regarding the specific diagnosis. For example, patients may differ in depression level regarding to the diagnosis. It should be explained, why Authors of the manuscript did not expect and measure any differences in stoic attitude among patient with different diagnosis.<br>Analyzing the differences in depression and coping strategies among cancer patients is very interesting, but it is not the objective of our study.<br>In the article there are some grammatical mistakes which need to be corrected, such as: p. 5 "Gender and stoicism differences on age, perception of life expectancy, and risk of toxicity, and psychological scales were measures using independent samples t-tests." - should be ..."were measured". p. 8: "In our study, social support, optimism, and coping based on positive attitude explined less stoicism in men." - should be: "... explained...".<br>Following the reviewer's indications, we have checked the text in detail and corrected the two spelling mistakes and one other error that we have detected.<br>We have highlighted them in blue in the text.<br><br>Cognizant of the contribution their insightful and critical observations have made to enhancing the quality of the text, the researchers of this study would again like to thank the Editor and reviewers for their time and effort.
**Additional Information:**	
**Question**	**Response**
**Financial Disclosure**<br><br>Enter a financial disclosure statement that describes the sources of funding for the work included in this submission. Review the submission guidelines for detailed requirements. View published research articles from *PLOS ONE* for specific examples.<br><br>This statement is required for submission and **will appear in the published article** if the submission is accepted. Please make sure it is accurate.	This work was supported by the FSEOM-Onvida 2015 grant for Projects on Long Survivors and Quality of Life. The sponsor of this research has not participated in data collection, analysis, or interpretation, in writing the report, or in the decision to submit the article for publication.

**Unfunded studies**<br>Enter: *The author(s) received no specific funding for this work.*<br><br>**Funded studies**<br>Enter a statement with the following details:<br>• Initials of the authors who received each award<br>• Grant numbers awarded to each author<br>• The full name of each funder<br>• URL of each funder website<br>• Did the sponsors or funders play any role in the study design, data collection and analysis, decision to publish, or preparation of the manuscript?<br>• **NO** - Include this sentence at the end of your statement: *The funders had no role in study design, data collection and analysis, decision to publish, or preparation of the manuscript.*<br>• **YES** - Specify the role(s) played.<br><br>* typeset	
**Competing Interests**<br><br>Use the instructions below to enter a competing interest statement for this submission. On behalf of all authors, disclose any competing interests that could be perceived to bias this work—acknowledging all financial support and any other relevant financial or non-financial competing interests.<br><br>This statement **will appear in the published article** if the submission is accepted. Please make sure it is accurate. View published research articles from *PLOS ONE* for specific examples.	he authors have declared that no competing interests exist.

**NO authors have competing interests**<br><br>Enter: *The authors have declared that no competing interests exist.*<br><br>**Authors with competing interests**<br><br>Enter competing interest details beginning with this statement:<br><br>*I have read the journal's policy and the authors of this manuscript have the following competing interests: [insert competing interests here]*<br><br>* typeset	
**Ethics Statement**<br><br>Enter an ethics statement for this submission. This statement is required if the study involved:<br><br>• Human participants<br>• Human specimens or tissue<br>• Vertebrate animals or cephalopods<br>• Vertebrate embryos or tissues<br>• Field research<br><br>Write "N/A" if the submission does not require an ethics statement.<br><br>General guidance is provided below. Consult the submission guidelines for detailed instructions. **Make sure that all information entered here is included in the Methods section of the manuscript.**	Conflicts of interest<br>The authors have declared no conflict of interest related to this study.<br>Ethics approval<br>The study was approved by the Research Ethics Committee of the Principality of Asturias (19 January 2015) and by the Spanish Agency of Medicines and Medical Devices (AEMPS) (numberto: L34LM-MM2GH-Y925U-RJDHQ).<br>Research involving Human Participants<br>The study has been performed in accordance with the ethical standards of the 1964 Declaration of Helsinki and its later amendments. This study is an observational, non-interventionist trial.<br>Consent to participate and for publication<br>Signed informed consent was obtained from all patients.<br>Consent for publication<br>Informed consent and approval by the national competent authorities includes permission for publication and diffusion of the data.

**Format for specific study types**<br><br>**Human Subject Research (involving human participants and/or tissue)**<br>• Give the name of the institutional review board or ethics committee that approved the study<br>• Include the approval number and/or a statement indicating approval of this research<br>• Indicate the form of consent obtained (written/oral) or the reason that consent was not obtained (e.g. the data were analyzed anonymously)<br><br>**Animal Research (involving vertebrate animals, embryos or tissues)**<br>• Provide the name of the Institutional Animal Care and Use Committee (IACUC) or other relevant ethics board that reviewed the study protocol, and indicate whether they approved this research or granted a formal waiver of ethical approval<br>• Include an approval number if one was obtained<br>• If the study involved *non-human primates*, add *additional details* about animal welfare and steps taken to ameliorate suffering<br>• If anesthesia, euthanasia, or any kind of animal sacrifice is part of the study, include briefly which substances and/or methods were applied<br><br>**Field Research**<br><br>Include the following details if this study involves the collection of plant, animal, or other materials from a natural setting:<br>• Field permit number<br>• Name of the institution or relevant body that granted permission	
**Data Availability**<br><br>Authors are required to make all data underlying the findings described fully available, without restriction, and from the time of publication. PLOS allows rare exceptions to address legal and ethical concerns. See the PLOS Data Policy and FAQ for detailed information.	No - some restrictions will apply

A Data Availability Statement describing where the data can be found is required at submission. Your answers to this question constitute the Data Availability Statement and **will be published in the article**, if accepted.<br><br>**Important:** Stating 'data available on request from the author' is not sufficient. If your data are only available upon request, select 'No' for the first question and explain your exceptional situation in the text box.<br><br>Do the authors confirm that all data underlying the findings described in their manuscript are fully available without restriction?	
**Describe where the data may be found in full sentences. If you are copying our sample text, replace any instances of XXX with the appropriate details.**<br><br>• If the data are **held or will be held in a public repository**, include URLs, accession numbers or DOIs. If this information will only be available after acceptance, indicate this by ticking the box below. For example: *All XXX files are available from the XXX database (accession number(s) XXX, XXX.*).<br>• If the data are all contained **within the manuscript and/or Supporting Information files**, enter the following: *All relevant data are within the manuscript and its Supporting Information files.*<br>• If neither of these applies but you are able to provide **details of access elsewhere**, with or without limitations, please do so. For example:<br><br>*Data cannot be shared publicly because of [XXX]. Data are available from the XXX Institutional Data Access / Ethics Committee (contact via XXX) for researchers who meet the criteria for access to confidential data.*<br><br>*The data underlying the results presented in the study are available from (include the name of the third party*	Availability of data and material (data transparency)<br>The database is available through a centralized web platform: www.neocoping.es.

and contact information or URL).<br>• This text is appropriate if the data are owned by a third party and authors do not have permission to share the data.<br><br>* typeset	
Additional data availability information:	Tick here if your circumstances are not covered by the questions above and you need the journal’s help to make your data available.

Powered by Editorial Manager® and ProduXion Manager® from Aries Systems Corporation

Manuscript

Click here to access/download;Manuscript;04022022 Stoicism article PlosOne.docx

Article type: Original article

Title: Stoic attitude in 932 patients with cancer from the NEOcoping study: cross-sectional study

Running Title: Stoic attitude in patients with cancer

Authors: David Gomez[1], Alberto Carmona-Bayonas[2], Raquel Hernandez[3], Oliver Higuera[4], Jacobo Rogado-Revuelta[5], Vilma Pacheco-Barcia[6], María Valero[7], Mireia Gil Raga[8], Mª Mar Muñoz[9], Rafael Carrión-Galindo[10], Paula Jimenez-Fonseca[11], Caterina Calderon[12]

Affiliations:
[1] Department of Medical Oncology. Hospital Universitario Central of Asturias, IPSA, Oviedo, Universidad del País Vasco (UPV/EHU), País Vasco, Spain. https://orcid.org/0000-0002-1930-9660
[2] Department of Hematology and Medical Oncology, Hospital Universitario Morales Meseguer, Instituto Murciano de Investigación Biosanitaria (IMIB), UMU, Murcia, Spain. https://orcid.org/0000-0002-7265-0251
[3] Department of Medical Oncology. Hospital Universitario de Canarias, Tenerife, Spain. https://orcid.org/0000-0003-3426-7515
[4] Department of Medical Oncology. Hospital Universitario La Paz, Madrid, Spain.
[5] Department of Medical Oncology. Hospital Universitario Infanta Leonor, Madrid, Spain. https://orcid.org/0000-0002-9795-8762
[6] Department of Medical Oncology. Hospital Central de la Defensa Gomez Ulla, Madrid, Spain.
[7] Department of Medical Oncology. Hospital Quirón salud Sagrado Corazón, Sevilla, Spain. https://orcid.org/0000-0002-2539-5710
[8] Department of Medical Oncology. Consorcio Hospital General Universitario de Valencia, Valencia, Spain.
[9] Department of Medical Oncology. Hospital Virgen de La Luz, Cuenca, Spain. https://orcid.org/0000-0003-2579-8947
[10] Department of Medical Oncology. Hospital Universitario del Sureste, Arganda del Rey-Madrid, Spain.
[11] Department of Medical Oncology. Hospital Universitario Central of Asturias, IPSA, Oviedo, Universidad del País Vasco (UPV/EHU), País Vasco, Spain. https://orcid.org/0000-0003-4592-3813
[12] Department of Clinical sychology and Psychobiology. Faculty of Psychology. University of Barcelona, Barcelona, University of País Vasco (UPV/EHU), Pais Vasco, Spain. https://orcid.org/0000-0002-6956-9321

Corresponding author: Caterina Calderon, PhD. Department of Clinical Psychology and Psychobiology. Faculty of Psychology. University of Barcelona, Spain. Postal address: Passeig de la Vall d'Hebron, 171. 08035 Barcelona, Spain. Email address: ccalderon@ub.dedu

Abstract

Aim: The aim was to explore stoicism in patients with cancer, and to assess its correlation with coping, psychological distress, optimism, perceived social support, and estimated risk of recurrence and toxicity.

Method: NEOcoping is a multicenter, cross-sectional study. Participants' data were collected using a standardized, self-report form and LSS, MSPSS, Mini-MAC, BSI-18, and LOT-R questionnaires. Linear regression analyses were used to assess the association between stoicism and distress scores in both genders. A total of 932 individuals with non-metastatic, resected cancer were recruited.

Results: Males perceived a higher risk of recurrence and toxicity with adjuvant chemotherapy and obtained higher stoic attitude scores than females. Women scored higher on somatization, depression, and anxiety. Patients with high stoicism scores were older and experienced more maladaptive coping (helplessness, anxious preoccupation), and depression, while those with lower stoicism scores had greater perceived social support, positive attitude, and optimism. In both males and females, stoicism correlated negatively with perceived social support, positive attitude, and optimism and positively with helplessness, anxious preoccupation, and depression. In men, stoicism also correlated positively with a perception of a higher risk of recurrence and with anxiety, and in women, with age. Of the variance in stoicism, 15.6% was accounted by perceived social support, optimism, and anxious preoccupation in men and 16.9% was explained by perceived social support, optimism and age in women.

Discussion: Stoic attitude was associated with worse coping, greater psychological distress, decreased optimism, and perception of less social support in this series of patients with cancer.

Keywords: Brief Symptom Inventory, Liverpool Stoicism Scale, Mental Adjustment to Cancer, Multidimensional scale of perceived social support, Revised Life Orientation Test, Stoicism

Introduction

Stoicism, the school of philosophy created by Zeno of Citium (332-263 BC), is one of the philosophical doctrines with the most rebirths throughout history, influencing numerous modern, psychological, humanistic, and moral currents of thought [1]. Originally, stoicism sought a balanced solution to the dichotomy of determinism-free will, advocating a virtue that resided in knowledge of the laws of the cosmos and pragmatic acceptance of them. However, in its modern psychological conception, stoicism has come to denote a construct based on the suppression of all emotional expression, increased tolerance for suffering, and the acceptance of the vicissitudes of life without complaint [2].

Despite the potential explanatory capacity of different attitudes, stoicism has been ignored in health-related spheres [1]. One of the causes lies in the cultural and personality-based parameters that define stoicism. For Spiers, stoic traits would be adaptive in the area of health, as they would increase self-efficacy by perceiving one's own limits and prompting the adoption of proactive measures to control symptoms through the use of reason [3]. In contrast, according to other researchers, stoic characteristics hinder seeking care, delay diagnosis, and prevent the implementation of improvements in psychological care [1].

In oncology, an issue worth exploring is whether a stoic attitude has an influence, not only in terminal disease [4–6], but also in the early stages of localized, potentially curable cancers. The primary aim of this research, therefore, was to explore associations of stoicism with other outcomes in patients with cancer. Stoicism has been associated with being male, and Wagstaff and Rowledge found that men exhibited higher stoicism scores; nevertheless, no studies on the associations with stoicism specifically in men and women with cancer [2]. Our second objective was to assess gender differences in age, perception of the risk of cancer relapse, and the risk of adjuvant chemotherapy-related toxicity. Finally, we have developed a model of stoicism correlating with coping, psychological stress, optimism, and perceived social support.

Methods

Study design and population

NEOcoping is a cross-sectional, observational, and multicenter study sponsored by the Continuous Care Group of the Spanish Society of Medical Oncology (SEOM). Patients were recruited consecutively in eleven Spanish university hospitals between 2017 and 2019. After providing written informed consent, eligible patients were included during their first visit to a medical oncology department, which they attended in the month following resection of non-metastatic cancer. The structure of the first visit followed the standard practice of each center, requiring, at the very least, that the diagnosis, prognosis or risk of cancer recurrence, and therapeutic alternatives, with their

incumbent risks of toxicity and benefits be explained. Once the decision on whether or not to receive chemotherapy was made, the subject was invited to participate in this study, making the decision to accept systemic adjuvant treatment completely independent of recruitment. The ethics review board at each of the participating institutions and the Spanish Agency for Medicines and Medical Devices (AEMPS) approved the study and all procedures were in accordance with the Helsinki Declaration.
Inclusion criteria were ≥18 years of age with resected, non-metastatic cancer for which the clinical practice guidelines recommended adjuvant chemotherapy. Exclusion criteria were cognitive impairment, any neoadjuvant therapy, adjuvant treatment consisting solely of radiotherapy, hormone therapy or other treatment modalities other than chemotherapy, any contraindication for adjuvant chemotherapy (poor functional status, age, comorbidities, etc.). Of the 1.016 patients screened, 84 were not eligible (23 did not meet inclusion criteria, 28 met exclusion criteria, and data were missing for 33). A total of 932 individuals with non-metastatic, resected cancer were recruited.

Instruments and data collection

Data were collected from the individuals and from clinical records. Psychometric questionnaires were completed by the patients at home and delivered at the next medical visit during which the oncologist clarified any doubts surrounding the questions in the questionnaires. Participants were informed that the answers would be treated anonymously and that there were no "right" or "wrong" answers, with the aim of minimizing the risk of random response. Data were collected through the centralized web platform www.neocoping.es that controls for lost data, inconsistencies, and errors in real time, and double-checks the study variables, with telephone and on-line monitoring (PJF).

Liverpool Stoicism Scale (LSS) is a self-report questionnaire designed to measure stoicism; it contains 20 items, including lack of emotional involvement, dislike for openly expressing emotion, and the ability to withstand emotion. The Spanish version was translated and validated by our group [7]. Responses were recorded on a 5-point Likert scale ranging from *strongly disagree* to *strongly agree,* yielding a sum score of between 20 and 100. Higher scores indicate greater stoicism. The scale has good internal consistency (Cronbach's alpha=0.83) [8].

Multidimensional scale of perceived social support (MSPSS) is a brief scale that assesses three sources of support: family, friends, and significant other. Items were measured on a 5-point Likert scale [9]. A higher score indicates more perceived support. The scale in this study has good internal consistency (Cronbach's alpha=0.90).

Mini-Mental Adjustment to Cancer (Mini-MAC) scale is one of the most widely used instruments to quantify coping responses in individuals with cancer [10]. The 29-item mini-MAC assesses five cognitive coping responses: helplessness (e.g., '*Can't handle it*'), anxious preoccupation (e.g., '*I am apprehensive*'), positive attitude (e.g., '*At the moment I take one day at a time*'), and cognitive avoidance (e.g., '*Not thinking about it helps me cope*'). Each item is scored from 1 to 4. High scores

indicate that coping styles are used more often. Satisfactory reliability was found for scales, Cronbach's alpha ranged from 0.52 to 0.90 [11].

Brief Symptom Inventory (BSI-18) includes 18 symptoms to evaluate the degree of distress on a 5-point Likert scale [12]. The scale groups symptoms into: somatization, depression, and anxiety. Cronbach's alpha ranged from 0.81 to 0.90 [13].

Revised Life Orientation Test (LOT-R) consists of optimism and pessimism scales, with ten items (three items assess optimism, three evaluate pessimism, and four are fillers). Respondents indicated the extent to which they agreed with each item on a 5-point Likert scale. In this study, Cronbach's alpha ranged between 0.74 and 0.78.

Patients were asked to predict their risk of cancer recurrence with and without chemotherapy, as well as their risk of severe toxicity with chemotherapy, using a numerical rating scale from 0 to 100.

Demographic data. The following data were obtained with respect to the sample's medical and demographic characteristics. Patient characteristics included gender; age; marital status; educational level; occupational field; tumor site, stage, and time since diagnosis. Clinical estimations were obtained from the medical oncologist who cared for the patient at their first appointment to assess the suitability of administering adjuvant treatment after surgery with curative intent.

Data analysis

Demographic information was reported by frequency analyses. Gender and stoicism differences on age, perception of life expectancy, and risk of toxicity, and psychological scales were measures using independent samples t-tests. Chi-squared analyses were used to investigate potential differences between men and women. Effect sizes were calculated by Cohen's statistics. Bivariate correlations were calculated between stoicism (LSS) and psychological scales (MSPSS, Mini-Mac, BSI-18, and LOT-R). All data were checked for normality, outliers, and the assumptions of multicollinearity and homocedascity. While the psychological scales (LSS, MSPSS, Mini-MAC, BSI-18, and LOT-R) showed a discreet correlation, it was not high enough to suggest problems with multicollinearity. Two linear regression analyses were used to predict the association between stoicism and psychological scales in males and females. In the subsequent statistical analyses, stoicism served as the criterion variable in the regression analyses, while social support (as per the MSPSS), coping strategies (Mini-MAC), psychological distress (BSI-18), and optimism (LOT-R) were utilized as predictors. For all the tests carried out, bilateral statistical significance was set at $p<.05$. Statistical analyses were performed using the IBM-SPSS 23.0 statistical software package for Windows PC.

Results

Patients' baseline characteristics

Mean age was 62.1 years (standard deviation [SD]=12.1, range 24-85) and 61% were female. Baseline characteristics are presented in **Table 1**. The most frequent tumor types were adenocarcinomas of the colon (42%), breast (34.4%), and stomach (4.5%). Approximately half (43.8%) were stage III. Most subjects were married or living with a partner (76.3%), had at least primary education (53.6%), and claimed to be unemployed or retired (58.0%).

The estimated risk of relapse with chemotherapy was 21.1%, and 37.7% without chemotherapy.

- Insert here Table 1-

Gender differences with respect to age, perception of risk of recurrence and toxicity, stoicism, and psychological scales in patients with cancer

The men in this sample were older (t=6.549; p<0.001) and estimated a higher risk of recurrence without chemotherapy (t=2.711; p=0.007) and lower risk of toxicity with chemotherapy (t=-3.717; p=0.001) than females. No differences were found between the two genders in the perception of the risk of recurrence with chemotherapy, **Table 2**.

Males scored higher for stoic attitude than women (t=8.388; p<0.001). Women, on the other hand, scored higher for somatization, depression and anxiety (t=-4.547, t=-5.802 and t=-3.002; all p<0.001), as well as for anxious preoccupation (t=-3.554; p<0.001). The remaining differences were not statistically significant (p>0.05).

- Insert here table 2-

Stoicism differences by age, perception of risk of recurrence and toxicity, and psychological scales in patients with cancer

Of the 932 subjects, 33.5% (n=312) scored high in stoicism (percentile>75), and 26.8% (n=250) low (percentile<25) on the LSS. Patients with high stoicism scores were older than those who scored lower on this domain; mean age 64.4 versus 61.4 years, respectively (t=-6.941; p<0.001), and more depressed (t=-2.609; p=0.009) **Table 3**. As for individuals' estimation of the risk of relapse and toxicity with chemotherapy based on their level of stoicism, no statistically significant differences were found.

Participants with lower stoicism scores had greater perceived social support, positive attitude, and higher levels of optimism than more stoic individuals. On the other hand, subjects with high stoicism scores had more maladaptive coping based on helplessness and anxious preoccupation than patients with low scores. The remaining differences between the two groups were not statistically significant, **Table 3**.

- Insert here table 3-

Relation between stoicism and social and psychological scales in men and women

Correlations were calculated between stoicism and the main study variables in men and women (**Table 4**). Bivariate correlations between LSS and psychological scales were inconsistent with the notion of stoicism as a factor in psychological resilience. In males, stoicism correlated negatively with perceived social support, coping strategy (positive attitude), and optimism, and positively with maladaptive coping (helplessness and anxious preoccupation), anxiety, depression and estimation of the risk of recurrence without chemotherapy. In women, stoicism correlated negatively with perceived social support, positive attitude and optimism, and positively with age, coping strategies (helplessness, and anxious preoccupation), and depression.

- Insert here table 4-

Explanatory model of stoicism in people with cancer

Table 5 presents the results of the linear regression analyses performed to explain stoicism in patients with resected, non-metastatic cancer by gender. Structural equation models were then conducted separately for males and females. In men, the results of the linear regression indicated that 15.6% of the variance in stoicism (F =21.738; p=<0.001) was accounted for by perceived social support (MSPSS), optimism (LOT-R), and anxious preoccupation (Mini-MAC scale). In women, 16.9% of the variance in stoicism (F=35.622; $p \leq 0.001$) was explained by perceived social support (MSPSS), optimism (LOT-R), and age.

- Insert here table 5-

Discussion

With this study, we have explored the influence of stoic traits on coping, perceived social support, psychological status, optimism, and estimation of risk of relapse and chemotherapy toxicity in people with resected, non-metastatic cancer prior to the initiation of adjuvancy. Moreover, we have examined these traits by gender. Half of the sample exhibited stoic attitudes — more so in men than in women. Stoicism was seen to be more of a risk factor than a protective one, since more stoic individuals perceived less social support, used passive coping strategies, and displayed worse psychological status with greater anxiety and depression. This led the high stoicism-scoring participants to adapt worse to their disease. These results endorse the notion of Pinnock et al., who pointed out that stoicism could potentially be a maladaptive behavior with negative attitudes toward seeking psychological help [14].

In line with earlier studies, stoicism and psychological suffering were more common in males than women [1,2,14–16]. In our series, the males were older, with little age dispersion [mean age, 62.1; mode, 68]; colon was the most frequent cancer. In contrast, among the females, there were two age

groups, a younger one in which breast cancer was the most common tumor site and another, older group [mean age, 56.9; mode, 48] with predominance of breast and colon cancer. As such, stoicism was unrelated to age in the men insofar as most of them were older, whereas age did impact stoicism in women, younger females were less stoic than older ones, as reflected in the literature [1]. It is possible that stoicism increases with development in adults and age-related maturity, given the acceptance that loss is inevitable. Likewise, some authors have explained it as a cohort effect; i.e., that older people have grown up in a culture in which giving up, resignation, and stoic attitudes are particularly valued [1]. Similarly, some authors suggest that males are more stoic because it is more difficult for them to identify and express their emotions or that they are afraid of appearing weak and 'unmanly' in light of their illness [1,17,18].

Despite these discrepancies, there are more similarities than differences between men and women who have high stoicism scores. The most salient of them being that they express having less social support and are more pessimistic. Thus, it may be that if the stoic does not articulate what is happening to them very much, the people around them adapt to this style and ask less so as 'not to bother them' and the patient perceives it as a lack of support.

We did not detect any relationship between stoicism and the perception of risk of relapse or of toxicity. Nevertheless, there were differences between the genders, inasmuch as males perceived a greater risk of relapse without chemotherapy that might be associated with their greater pessimism in facing cancer. Likewise, males exhibited less anxiety and depression than females. A classic study conducted by Pettingale KW et al. in females with early stage breast cancer found that stoicism was associated with less stoic acceptance and coping based on positive attitude with longer survival [19]. In our study, social support, optimism, and coping based on positive attitude explained less stoicism in men. Stoic individuals who perceive less social support, are less optimistic, and display a passive style of coping with illness, tend to be more withdrawn — all related with worse adjustment to their disease. In contrast, Behen JM and Rodriguez JR found no correlation between resignation, social support, and coping with illness [20]. A review of eleven studies concluded that the experience of living with cancer in old age is characterized by profound ambiguity and a predominance of social withdrawal [21]. In women, stoicism was more common in older aged individuals [1] and was associated with less perceived social support and lower levels of optimism. In line with our series, Schou I et al. found a correlation between pessimism as quantified by the LOT-R scale, maladaptive coping, and depression in their study of women with breast cancer conducted one year following surgery [22].

This study has certain limitations. First of all, given its cross-sectional design, a causal interpretation cannot be made of the association between stoicism and psychological variables. Stoic perspective could only be assessed at a given point in time and not throughout the entire process of adapting to

cancer. Secondly, we have evaluated patients' subjective appraisal of their social network without estimating the actual amount of support they received or the support perceived by the family. Thirdly, stoicism and psychological traits were not evaluated prior to the diagnosis of cancer; as a result, the impact cancer had on them cannot be estimated.

In conclusion, stoicism was a factor that was associated with worse adaptation to cancer and was more common in males and in older participants. A stoic attitude correlated with perception of less social support, decreased optimism, passive coping strategies (helplessness and anxious preoccupation), and greater psychological distress (depression).

Declarations

Conflicts of interest

The authors have declared no conflict of interest related to this study.

Ethics approval

The study was approved by the Research Ethics Committee of the Principality of Asturias (19 January 2015) and by the Spanish Agency of Medicines and Medical Devices (AEMPS) (numberto: L34LM-MM2GH-Y925U-RJDHQ).

Research involving Human Participants

The study has been performed in accordance with the ethical standards of the 1964 Declaration of Helsinki and its later amendments. This study is an observational, non-interventionist trial.

Consent to participate and for publication

Signed informed consent was obtained from all patients.

Consent for publication

Informed consent and approval by the national competent authorities includes permission for publication and diffusion of the data.

Availability of data and material (data transparency)

The database is available through a centralized web platform: www.neocoping.es.

Code availability

Patients are identified by an encrypted code known only to the local researcher.

Acknowledgements

The authors would like to thank the investigators of the NEOcoping study, the Supportive Care Working Group of the Spanish Society of Medical Oncology (SEOM), Priscilla Chase Duran for editing the manuscript, and Natalia Cateriano, Miguel Vaquero, and IRICOM S.A. for the support of the website registry.

Authors' contributions

A.C.B., P.J.F. and C.C. developed the project, analyzed the data and drafted the manuscript. The other authors recruited patients and provided clinical information, comments, and improvements to the manuscript. All authors participated in the interpretation and discussion of data, and the critical review of the manuscript.

References

1. Moore A, Grime J, Campbell P, Richardson J (2013). Troubling stoicism: Sociocultural influences and applications to health and illness behaviour. Health 17:159–173.
2. Wagstaff GF, Rowledge AM (1995). Stoicism: Its relation to gender, attitudes toward poverty, and reactions to emotive material. J Soc Psychol 135:181–184.
3. Spiers J (2006). Expressing and responding to pain and stoicism in home- care nurse–patient interactions. Scand J Caring Sci 20:293–301.
4. Garro LC (1990). Culture, pain and cancer. J Palliat Care 6:34-44.
5. Hillier R (1990). Control of pain in terminal cancer. Br Med Bull 46:279–291.
6. Hinton J (1994). Which patients with terminal cancer are admitted from home care? Palliat Med 8:197–210.
7. Calderon C, Ferrando PJ, Lorenzo-Seva U, Carmona-Bayonas A, Jara C, Ayala de la peña F, Beato C, Ramchandani A, García T, Mangas-Izquierdo M, Castelo B, Donnay O, Muñoz MM, Martínez de Castro E, García S, Ghanem I, Mellen T, Jiménez-Fonseca P (2017). Psychometric properties of Liverpool Stoicism Scale (LSS) in a cohort of patients with resected cancer in adjuvant treatment. Ann Psych. 2017;33:621.
8. Zimet GD, Powell SS, Farley GK, Werkman S, Berkoff KA (1990). Psychometric characteristics of the Multidimensional Scale of Perceived Social Support. J Pers Assess 55:610–617.
9. Vaillo YA, Pérez SM, López PM, Retes RR (2018). Mini-Mental Adjustment to Cancer Scale: Construct validation in Spanish breast cancer patients. J Psychosom Res 114:38–44.
10. Bredal IS (2010). The Norwegian version of the Mini- Mental Adjustment to Cancer Scale: factor structure and psychometric properties. Psycho- Oncol 19:216–221.
11. Galdón MJ, Durá E, Andreu Y, Ferrando M, Murgui S, Pérez S, Ibañez E (2008). Psychometric properties of the Brief Symptom Inventory-18 in a Spanish breast cancer sample. J Psychosom Res 65:533–539.
12. Cano-García FJ, Sanduvete-Chaves S, Chacón-Moscoso S, Rodríguez-Franco L, García-Martínez J, Antuña-Bellerín MA, Pérez-Gil JA (2015). Factor structure of the Spanish version of the Life Orientation Test-Revised (LOT-R): Testing several models. Int J Clin Heal Psychol 15:139–148.
13. Merckaert I, Libert Y, Messin S, Milani M, Slachmuylder J, Razavi D (2010). Cancer patients' desire for psychological support: prevalence and implications for screening patients' psychological needs. Psycho- Oncol 19:141–9.
14. Veloso AG, Sperling C, Holm LV, Nicolaisen A, Rottmann N, Thayssen S, Christensen RD, Knudsen JL, Hansen DG (2013). Unmet needs in cancer rehabilitation during the early cancer trajectory–a nationwide patient survey. Acta Oncol 52:372–381.

15. Burg MA, Adorno G, Lopez EDS, Loerzel V, Stein K, Wallace C, Sharma DKB (2015). Current unmet needs of cancer survivors: Analysis of open- ended responses to the American Cancer Society Study of Cancer Survivors II. Cancer 121:623–630.
16. Addis ME, Mahalik JR (2003). Men, masculinity, and the contexts of help seeking. Am Psychol 58:5.
17. Sobralske M (2006). Machismo sustains health and illness beliefs of Mexican American men. J Am Acad Nurse Pract 18:348–350.
18. Pettingale K, Morris T, Greer S, Haybittle JL (1985). Mental attitudes to cancer: an additional prognostic factor. Lancet 325:750.
19. Behen JM, Rodrigue JR (1994). Predictors of coping strategies among adults with cancer. Psychol Rep 74:43–48.
20. Hughes N, Closs SJ, Clark D (2009). Experiencing cancer in old age: a qualitative systematic review. Qual Health Res 19:1139–1153.
21. Schou I, Ekeberg Ø, Ruland CM, Sandvik L, Kåresen R (2004). Pessimism as a predictor of emotional morbidity one year following breast cancer surgery. Psycho- Oncol 13:309–320.

TABLES (5)

Table 1. Patient characteristics (n =932)

Characteristic	N	%
Gender		
Female	566	60.7
Males	366	39.3
Age, years		
Less than 45	144	15.5
46 – 55	203	21.8
56 – 65	265	28.4
More than 66	320	34.3
Cancer type		
Colon	391	42.0
Breast	321	34.4
Stomach	42	4.5
Others	178	19.1
Cancer stage		
I	130	14.0
II	390	41.9
III	408	43.8
Missing	3	0.3
Educational level		
Less than high school	500	53.6
More than high school	432	46.4
Marital status		
Married/ partnered	710	76.3
Single	80	8.4
Separated/ divorced	79	8.5
Widowed	63	6.8
Employment status		
Full or part-time	540	58.0
Retired, homemaker, unemployed	392	42.0

Table 2. Gender differences in age, perception of risk of recurrence and toxicity, stoicism, and psychological scales in patients with cancer

	Men (n=366)		Women (n=566)		t-value	
**Variables**	**Mean**	**SD**	**Mean**	**SD**	**T**	**p**
Age (years)	62.1	12.1	56.9	11.8	6.549	0.001*
Perception of risk of:						
Recurrence with CT	78.6	22.1	78.1	21.6	0.320	---
Recurrence without CT	36.7	21.4	32.4	22.5	2.711	0.007*
CT-related toxicity	53.7	22.7	59.4	22.4	-3.717	0.001*
LSS. Stoicism	58.4	7.2	54.2	7.7	8.388	0.001*
MSPSS. Social support	74.7	9.3	75.7	8.9	-1.634	---
Mini-MAC. Helplessness	19.8	19.1	19.2	19.5	0.536	---
Mini-MAC. Anxious preoccupation	36.7	21.6	42.2	23.6	-3.554	0.001*
Mini-MAC. Positive attitude	74.5	17.1	76.1	15.7	-1.404	---
Mini-MAC. Cognitive avoidance	51.5	26.3	54.8	26.0	-1.887	---
BSI-18. Somatization	59.7	6.7	61.8	6.9	-4.547	0.001*
BSI-18. Depression	59.2	6.7	61.5	6.0	-5.802	0.001*
BSI-18. Anxiety	59.7	7.0	63.0	6.9	-7.384	0.001*
LOT-R. Optimism	20.7	3.7	21.1	4.2	-1.478	---

Abbreviations: SD, Standard Deviation; CT, chemotherapy; LSS, Liverpool Stoicism Scale; MSPSS, Multidimensional Scale of Perceived Social Support; Mini-MAC, Mini-Mental Adjustment to Cancer; BSI, Brief Symptom Inventory; LOT-R, Life Orientation Test-Revised
* Statistically significant differences

Table 3. Comparison of cancer patients with high and low stoicism by age, perception of risk of recurrence and toxicity, and psychological scales

	**Low stoicism (PC <25) n =250**		**High stoicism (PC >75) n =312**		**t-test for equality of means[a]**	
**Variables**	Mean	SD	Mean	SD	t	P
Age (years)	64.4	12.1	61.4	11.6	-6.941	0.001*
Perception of risk of:						
Recurrence with CT	78.2	21.4	78.9	22.1	-0.412	---
Recurrence without CT	32.9	23.3	36.8	23.6	-1.932	---
Toxicity with CT	58.7	23.1	57.4	23.0	0.673	---
MSPSS. Social support	78.7	6.4	71.9	10.6	8.857	0.001*
Mini-MAC. Helplessness	13.8	16.9	20.8	19.9	-4.393	0.001*
Mini-MAC. Anxious preoccupation	39.1	22.6	42.4	24.5	-2.610	0.009*
Mini-MAC. Positive attitude	78.6	14.8	70.9	17.8	5.498	0.001*
Mini-MAC. Cognitive avoidance	51.6	26.8	52.3	26.1	-0.325	---
BSI-18. Somatization	61.6	6.9	61.3	7.2	0.487	---
BSI-18. Depression	60.2	5.5	61.6	6.5	-2.609	0.009*
BSI-18. Anxiety	62.1	7.7	62.3	8.3	-0.229	---
LOT-R. Optimism	22.1	4.2	20.0	3.9	5.990	0.001*

Abbreviation: PC, percentile, SD, Standard Deviation; CT, chemotherapy; LSS, Liverpool Stoicism Scale; MSPSS, Multidimensional Scale of Perceived Social Support; Mini-MAC, Mini-Mental Adjustment to Cancer; BSI, Brief Symptom Inventory; LOT-R, Life Orientation Test-Revised

[a] Before t-test analysis, Levene's test for equality of variances was applied.

* Statistically significant differences

Table 4. Correlation between stoicism, perception of risk of recurrence and toxicity, psychological scales in men and women with cancer

	Men		Women	
**Variables****	***r***	***p***	***r***	***p***
Age (years)	0.044	0.398	0.261	0.001*
Perception of risk of:				
Recurrence with CT	-0.031	---	0.020	---
Recurrence without CT	0.139	0.008*	-0.050	---
Toxicity with CT	-0.051	---	-0.030	---
MSPSS. Social support	-0.304	0.001*	-0.286	0.001*
Mini-MAC. Helplessness	0.130	0.013*	0.189	0.001*
Mini-MAC. Anxious preoccupation	0.199	0.001*	0.101	0.018*
Mini-MAC. Positive attitude	-0.198	0.001*	-0.149	0.038*
Mini-MAC. Cognitive avoidance	0.018	---	0.079	---
BSI-18. Somatization	0.041	---	0.012	---
BSI-18. Depression	0.176	0.001*	0.144	0.001*
BSI-18. Anxiety	0.133	0.011*	0.052	---
LOT-R. Optimism	-0.276	0.001*	-0.257	0.001*

Abbreviations: SD, Standard Deviation; LSS, Liverpool Stoicism Scale; MSPSS, Multidimensional Scale of Perceived Social Support; Mini-MAC, Mini-Mental Adjustment to Cancer; BSI, Brief Symptom Inventory; LOT-R, Life Orientation Test-Revised

* Statistically significant differences

**Criterion variable: stoicism according to LSS scale

Table 5. Summary of the linear regression analysis predicting for stoicism; corrected R (R^2), non-standardized coefficient (B), standard error, and standardized beta coefficient (β); analysis of variance and significance level

							95% IC	
**Predictor**	**R^2**	**B**	**SE**	**β**	**t**	***p***	**Lower**	**Upper**
**Men**								
(Constant)		78.171	3.308		23.634	0.001	71.666	84.676
Social Support[1]	15.6	-0.192	0.040	-0.246	-4.833	0.001	-0.270	-0.114
Optimism[2]		-0.348	0.101	-0.180	-3.440	0.001	-0.546	-0.149
Anxious preoccupation[3]		0.049	0.017	-0.145	2.886	0.004	0.015	0.082
**Women**								
(Constant)		67.399	3.272		20.600	0.001	60.972	73.826
Social support[1]	16.9	-0.189	0.035	-0.218	-5.273	0.001	-0.256	-0.117
Age		0.145	0.026	0.223	5.584	0.001	0.094	0.196
Optimism[2]		-0.349	0.075	-0.190	-4.634	0.001	-0.497	-0.201

Abbreviations: R^2, corrected R; B, non-standardized coefficient; SE, standard error; β, standard beta coefficient; t, analysis of variance; p, significance level, IC, interval confidence; LSS, Liverpool Stoicism Scale; MSPSS, Multidimensional Scale of Perceived Social Support; Mini-MAC, Mini-Mental Adjustment to Cancer; LOT-R, Life Orientation Test-Revised
[1]MSPSS. [2]LOT-R. [3]Mini-MAC subscale.
Criterion variable: stoicism according to LSS

Article type: Original article

Title: Stoic attitude in patients with cancer from the NEOcoping study: cross-sectional study

Running Title: Stoic attitude in patients with cancer

Authors: David Gomez[1], Alberto Carmona-Bayonas[2], Raquel Hernandez[3], Oliver Higuera[4], Jacobo Rogado-Revuelta[5], Vilma Pacheco-Barcia[6], María Valero[7], Mireia Gil Raga[8], Mª Mar Muñoz[9], Rafael Carrión-Galindo[10], Paula Jimenez-Fonseca[11], Caterina Calderon[12]

Affiliations:
[1] Department of Medical Oncology. Hospital Universitario Central of Asturias, IPSA, Oviedo, Universidad del País Vasco (UPV/EHU), País Vasco, Spain. https://orcid.org/0000-0002-1930-9660
[2] Department of Hematology and Medical Oncology, Hospital Universitario Morales Meseguer, Instituto Murciano de Investigación Biosanitaria (IMIB), UMU, Murcia, Spain. https://orcid.org/0000-0002-7265-0251
[3] Department of Medical Oncology. Hospital Universitario de Canarias, Tenerife, Spain. https://orcid.org/0000-0003-3426-7515
[4] Department of Medical Oncology. Hospital Universitario La Paz, Madrid, Spain.
[5] Department of Medical Oncology. Hospital Universitario Infanta Leonor, Madrid, Spain. https://orcid.org/0000-0002-9795-8762
[6] Department of Medical Oncology. Hospital Central de la Defensa Gomez Ulla, Madrid, Spain.
[7] Department of Medical Oncology. Hospital Quirón salud Sagrado Corazón, Sevilla, Spain. https://orcid.org/0000-0002-2539-5710
[8] Department of Medical Oncology. Consorcio Hospital General Universitario de Valencia, Valencia, Spain.
[9] Department of Medical Oncology. Hospital Virgen de La Luz, Cuenca, Spain. https://orcid.org/0000-0003-2579-8947
[10] Department of Medical Oncology. Hospital Universitario del Sureste, Arganda del Rey-Madrid, Spain.
[11] Department of Medical Oncology. Hospital Universitario Central of Asturias, IPSA, Oviedo, Universidad del País Vasco (UPV/EHU), País Vasco, Spain. https://orcid.org/0000-0003-4592-3813
[12] Department of Clinical sychology and Psychobiology. Faculty of Psychology. University of Barcelona, Barcelona, University of País Vasco (UPV/EHU), Pais Vasco, Spain. https://orcid.org/0000-0002-6956-9321

Corresponding author: Caterina Calderon, PhD. Department of Clinical Psychology and Psychobiology. Faculty of Psychology. University of Barcelona, Spain. Postal address: Passeig de la Vall d'Hebron, 171. 08035 Barcelona, Spain. Email address: ccalderon@ub.dedu

Abstract

Aim: The aim was to explore stoicism in patients with cancer, and to assess its correlation with coping, psychological distress, optimism, perceived social support, and estimated risk of recurrence and toxicity.

Method: NEOcoping is a multicenter, cross-sectional study. Participants' data were collected using a standardized, self-report form and LSS, MSPSS, Mini-MAC, BSI-18, and LOT-R questionnaires. Linear regression analyses were used to assess the association between stoicism and distress scores in both genders. A total of 932 individuals with non-metastatic, resected cancer were recruited.

Results: Males perceived a higher risk of recurrence and toxicity with adjuvant chemotherapy and obtained higher stoic attitude scores than females. Women scored higher on somatization, depression, and anxiety. Patients with high stoicism scores were older and experienced more maladaptive coping (helplessness, anxious preoccupation), and depression, while those with lower stoicism scores had greater perceived social support, positive attitude, and optimism. In both males and females, stoicism correlated negatively with perceived social support, positive attitude, and optimism and positively with helplessness, anxious preoccupation, and depression. In men, stoicism also correlated positively with a perception of a higher risk of recurrence and with anxiety, and in women, with age. Of the variance in stoicism, 15.6% was accounted by perceived social support, optimism, and anxious preoccupation in men and 16.9% was explained by perceived social support, optimism and age in women.

Discussion: Stoic attitude was associated with worse coping, greater psychological distress, decreased optimism, and perception of less social support in this series of patients with cancer.

Keywords: Brief Symptom Inventory, Liverpool Stoicism Scale, Mental Adjustment to Cancer, Multidimensional scale of perceived social support, Revised Life Orientation Test, Stoicism

Introduction

Stoicism, the school of philosophy created by Zeno of Citium (332-263 BC), is one of the philosophical doctrines with the most rebirths throughout history, influencing numerous modern, psychological, humanistic, and moral currents of thought [1]. Originally, stoicism sought a balanced solution to the dichotomy of determinism-free will, advocating a virtue that resided in knowledge of the laws of the cosmos and pragmatic acceptance of them. However, in its modern psychological conception, stoicism has come to denote a construct based on the suppression of all emotional expression, increased tolerance for suffering, and the acceptance of the vicissitudes of life without complaint [2].

Despite the potential explanatory capacity of different attitudes, stoicism has been ignored in health-related spheres [1]. One of the causes lies in the cultural and personality-based parameters that define stoicism. For Spiers, stoic traits would be adaptive in the area of health, as they would increase self-efficacy by perceiving one's own limits and prompting the adoption of proactive measures to control symptoms through the use of reason [3]. In contrast, according to other researchers, stoic characteristics hinder seeking care, delay diagnosis, and prevent the implementation of improvements in psychological care [1].

In oncology, an issue worth exploring is whether a stoic attitude has an influence, not only in terminal disease [4–6], but also in the early stages of localized, potentially curable cancers. A study of men with prostate cancer of any stage suggests that stoicism is neither adaptive nor maladaptive for distress and quality of life outcomes and is not highly correlated with male self-esteem or psychological flexibility. The authors hypothesized that this may mean that men use stoicism to cope with prostate cancer in line with their own masculine values [7]. In another study by Mah et al., younger, and older patients with advanced cancer showed unique biopsychosocial correlates of pain-related stoicism and caution [8]. Cognitive avoidance of distressing thoughts was intrapersonal, associated with greater concealment, self-doubt, and reticence in the elderly. Older adults were more likely to regulate emotions, to be less expressive about negative events and avoidance of negative information in relation to a more stoic attitude.

As cancer is a stressful event associated with fear and prognostic uncertainty, it is common for patients to employ coping strategies to deal with it. Stoicism and emotional distress (anxiety, depression, and somatization) may interfere with coping by increasing anxious preoccupation [9]. Dispositional optimism may act as a resource that maintains a positive mood, provides a more flexible coping style to face with the inability to control stressful stimuli, and protects patients from the possible negative effects of cancer treatment and cancer itself in case of recurrence [10,11]. Likewise, social support, defined as the perception that others are willing to help, appears to operate similarly

to optimism and is a critical factor in decreasing negative psychological affect in individuals with cancer [12].

Therefore, the available literature on the impact of stoicism in patients with non-metastatic cancer is very limited. Hence, the aim of this research, therefore, was to explore associations of stoicism with other outcomes in patients with non-metastatic cancer. Stoicism has been associated with being male, and Wagstaff and Rowledge found that men exhibited higher stoicism scores; nevertheless, no studies on the associations with stoicism specifically in men and women with cancer [2]. Our second objective was to assess gender differences in age, perception of the risk of cancer relapse, the risk of adjuvant chemotherapy-related toxicity, coping, psychological stress, optimism, and perceived social support.

Methods

Study design and population

NEOcoping is a cross-sectional, observational, and multicenter study sponsored by the Continuous Care Group of the Spanish Society of Medical Oncology (SEOM). Patients were recruited consecutively in eleven Spanish university hospitals between 2017 and 2019. After providing written informed consent, eligible patients were included during their first visit to a medical oncology department, which they attended in the month following resection of non-metastatic cancer. The structure of the first visit followed the standard practice of each center, requiring, at the very least, that the diagnosis, prognosis or risk of cancer recurrence, and therapeutic alternatives, with their incumbent risks of toxicity and benefits be explained. Once the decision whether or not to receive chemotherapy was made, the subject was invited to participate in this study, making the decision to accept systemic adjuvant treatment completely independent of recruitment. The ethics review board at each of the participating institutions and the Spanish Agency for Medicines and Medical Devices (AEMPS) approved the study and all procedures were in accordance with the Helsinki Declaration.
Inclusion criteria were ≥18 years of age with resected, non-metastatic cancer for which the clinical practice guidelines recommended adjuvant chemotherapy. Exclusion criteria were cognitive impairment, any neoadjuvant therapy, adjuvant treatment consisting solely of radiotherapy, hormone therapy or other treatment modalities other than chemotherapy, any contraindication for adjuvant chemotherapy (poor functional status, age, comorbidities, etc.). Of the 1.016 patients screened, 84 were not eligible (23 did not meet inclusion criteria, 28 met exclusion criteria, and data were missing for 33). A total of 932 individuals with non-metastatic, resected cancer were recruited.

Instruments and data collection

Data were collected from the individuals and from clinical records. Psychometric questionnaires were completed by the patients at home and delivered at the next medical visit during which the

oncologist clarified any doubts surrounding the questions in the questionnaires. Participants were informed that the answers would be treated anonymously and that there were no "right" or "wrong" answers, with the aim of minimizing the risk of random response. Data were collected through the centralized web platform www.neocoping.es that controls for lost data, inconsistencies, and errors in real time, and double-checks the study variables, with telephone and on-line monitoring (PJF).

Liverpool Stoicism Scale (LSS) is a self-report questionnaire designed to measure stoicism; it contains 20 items, including lack of emotional involvement, dislike for openly expressing emotion, and the ability to withstand emotion. The Spanish version was translated and validated by our group [13]. Responses were recorded on a 5-point Likert scale ranging from *strongly disagree* to *strongly agree,* yielding a sum score of between 20 and 100. Higher scores indicate greater stoicism. The scale has good internal consistency (Cronbach's alpha=0.83) [14].

Multidimensional scale of perceived social support (MSPSS) is a brief scale that assesses three sources of support: family, friends, and significant other. Items were measured on a 5-point Likert scale [15]. A higher score indicates more perceived support. The scale in this study has good internal consistency (Cronbach's alpha=0.90).

Mini-Mental Adjustment to Cancer (Mini-MAC) scale is one of the most widely used instruments to quantify coping responses in individuals with cancer [16]. The 29-item mini-MAC assesses five cognitive coping responses: helplessness (e.g., '*Can't handle it*'), anxious preoccupation (e.g., '*I am apprehensive*'), fighting spirit, fatalism (e.g., '*At the moment I take one day at a time*'), and cognitive avoidance (e.g., '*Not thinking about it helps me cope*'). For this study, the version with a 4-factor structure validated in Spanish was used, grouping fighting spirit and fatalism into a single factor, positive attitude [17]. Each item is scored from 1 to 4. High scores indicate that coping styles are used more often. Satisfactory reliability was found for scales, Cronbach's alpha ranged from 0.52 to 0.90 [18].

Brief Symptom Inventory (BSI-18) includes 18 symptoms to evaluate the degree of distress on a 5-point Likert scale [19]. The scale groups symptoms into somatization, depression, and anxiety. Cronbach's alpha ranged from 0.81 to 0.90 [20].

Revised Life Orientation Test (LOT-R) consists of optimism and pessimism scales, with ten items (three items assess optimism, three evaluate pessimism, and four are fillers). Respondents indicated the extent to which they agreed with each item on a 5-point Likert scale. In this study, Cronbach's alpha ranged between 0.74 and 0.78.

Patients were asked to predict their risk of cancer recurrence with and without chemotherapy, as well as their risk of severe toxicity with chemotherapy, using a numerical rating scale from 0 to 100.

Demographic data. The following data were obtained with respect to the sample's medical and demographic characteristics. Patient characteristics included gender; age; marital status; educational

level; occupational field; tumor site, stage, and time since diagnosis. Clinical estimations were obtained from the medical oncologist who cared for the patient at their first appointment to assess the suitability of administering adjuvant treatment after surgery with curative intent.

Data analysis

Demographic information was reported by frequency analyses. Gender and stoicism differences on age, perception of life expectancy, and risk of toxicity, and psychological scales were measured using independent samples t-tests. Chi-squared analyses were used to investigate potential differences between men and women. Effect sizes were calculated by Cohen's statistics. Bivariate correlations were calculated between stoicism (LSS) and psychological scales (MSPSS, Mini-Mac, BSI-18, and LOT-R). All data were checked for normality, outliers, and the assumptions of multicollinearity and homoscedasticity. While the psychological scales (LSS, MSPSS, Mini-MAC, BSI-18, and LOT-R) showed a discreet correlation, it was not high enough to suggest problems with multicollinearity. Two linear regression analyses were used to predict the association between stoicism and psychological scales in males and females. In the subsequent statistical analyses, stoicism served as the criterion variable in the regression analyses, while social support (as per the MSPSS), coping strategies (Mini-MAC), psychological distress (BSI-18), and optimism (LOT-R) were utilized as predictors. For all the tests carried out, bilateral statistical significance was set at $p<.05$. Statistical analyses were performed using the IBM-SPSS 23.0 statistical software package for Windows PC.

Results

Patients' baseline characteristics

Mean age was 62.1 years (standard deviation [SD]=12.1, range 24-85) and 61% were female. Baseline characteristics are presented in **Table 1**. The most frequent tumor types were adenocarcinomas of the colon (42%), breast (34.4%), and stomach (4.5%). Approximately half (43.8%) were stage III. Most subjects were married or living with a partner (76.3%), had at least primary education (53.6%), and claimed to be unemployed or retired (58.0%).

The estimated risk of relapse with chemotherapy was 21.1%, and 37.7% without chemotherapy.

- Insert here Table 1-

Gender differences with respect to age, perception of risk of recurrence and toxicity, stoicism, and psychological scales in patients with cancer

The men in this sample were older ($t=6.549$; $p<0.001$) and estimated a higher risk of recurrence without chemotherapy ($t=2.711$; $p=0.007$) and lower risk of toxicity with chemotherapy ($t=-3.717$; $p=0.001$) than females. No differences were found between the two genders in the perception of the risk of recurrence with chemotherapy, **Table 2**.

Males scored higher for stoic attitude than women (t=8.388; p<0.001). Women, on the other hand, scored higher for somatization, depression and anxiety (t=-4.547, t=-5.802 and t=-3.002; all p<0.001), as well as for anxious preoccupation (t=-3.554; p<0.001). The remaining differences were not statistically significant (p>0.05).

- Insert here table 2-

Stoicism differences by age, perception of risk of recurrence and toxicity, and psychological scales in patients with cancer

Of the 932 subjects, 33.5% (n=312) scored high in stoicism (percentile>75), and 26.8% (n=250) low (percentile<25) on the LSS. Patients with high stoicism scores were older than those who scored lower on this domain; mean age 64.4 versus 61.4 years, respectively (t=-6.941; p<0.001), and more depressed (t=-2.609; p=0.009) **Table 3**. As for individuals' estimation of the risk of relapse and toxicity with chemotherapy based on their level of stoicism, no statistically significant differences were found. Participants with lower stoicism scores had greater perceived social support, positive attitude, and higher levels of optimism than more stoic individuals. On the other hand, subjects with high stoicism scores had more maladaptive coping based on helplessness and anxious preoccupation than patients with low scores. The remaining differences between the two groups were not statistically significant, **Table 3**.

- Insert here table 3-

Relation between stoicism and social and psychological scales in men and women

Correlations were calculated between stoicism and the main study variables in men and women (**Table 4**). Bivariate correlations between LSS and psychological scales were inconsistent with the notion of stoicism as a factor in psychological resilience. In males, stoicism correlated negatively with perceived social support, coping strategy (positive attitude), and optimism, and positively with maladaptive coping (helplessness and anxious preoccupation), anxiety, depression and estimation of the risk of recurrence without chemotherapy. In women, stoicism correlated negatively with perceived social support, positive attitude and optimism, and positively with age, coping strategies (helplessness, and anxious preoccupation), and depression.

- Insert here table 4-

Explanatory model of stoicism in people with cancer

Table 5 presents the results of the linear regression analyses performed to explain stoicism in patients with resected, non-metastatic cancer by gender. Structural equation models were then conducted separately for males and females. In men, the results of the linear regression indicated that 15.6% of

the variance in stoicism (F =21.738; p=<0.001) was accounted for by perceived social support (MSPSS), optimism (LOT-R), and anxious preoccupation (Mini-MAC scale). In women, 16.9% of the variance in stoicism (F=35.622; $p \leq 0.001$) was explained by perceived social support (MSPSS), optimism (LOT-R), and age.

- Insert here table 5-

Discussion

With this study, we have explored the influence of stoic traits on coping, perceived social support, psychological status, optimism, and estimation of risk of relapse and chemotherapy toxicity in people with resected, non-metastatic cancer prior to the initiation of adjuvancy. Moreover, we have examined these traits by gender. Half of the sample exhibited stoic attitudes — more so in men than in women. Stoicism was seen to be more of a risk factor than a protective one, since more stoic individuals perceived less social support, used passive coping strategies, and displayed worse psychological status with greater anxiety and depression. This led the high stoicism-scoring participants to adapt worse to their disease. These results endorse the notion of Pinnock et al., who pointed out that stoicism could potentially be a maladaptive behavior with negative attitudes toward seeking psychological help [21].

In line with earlier studies, stoicism and psychological suffering were more common in males than women [1,2,21–23]. In our series, the males were older, with little age dispersion [mean age, 62.1; mode, 68]; colon was the most frequent cancer. In contrast, among the females, there were two age groups, a younger one in which breast cancer was the most common tumor site and another, older group [mean age, 56.9; mode, 48] with predominance of breast and colon cancer. As such, stoicism was unrelated to age in the men insofar as most of them were older, whereas age did impact stoicism in women, younger females were less stoic than older ones, as reflected in the literature [1]. It is possible that stoicism increases with development in adults and age-related maturity, given the acceptance that loss is inevitable. Likewise, some authors have explained it as a cohort effect; i.e., that older people have grown up in a culture in which giving up, resignation, and stoic attitudes are particularly valued [1]. Similarly, some authors suggest that males are more stoic because it is more difficult for them to identify and express their emotions or that they are afraid of appearing weak and 'unmanly' in light of their illness [1,24,25].

Despite these discrepancies, there are more similarities than differences between men and women who have high stoicism scores. The most salient of them being that they express having less social support and are more pessimistic. Thus, it may be that if the stoic does not articulate what is happening to them very much, the people around them adapt to this style and ask less so as 'not to bother them' and the patient perceives it as a lack of support.

We did not detect any relationship between stoicism and the perception of risk of relapse or of toxicity. Nevertheless, there were differences between the genders, inasmuch as males perceived a greater risk of relapse without chemotherapy that might be associated with their greater pessimism in facing cancer. Likewise, males exhibited less anxiety and depression than females. A classic study conducted by Pettingale KW et al. in females with early stage breast cancer found that stoicism was associated with less stoic acceptance and coping based on positive attitude with longer survival [26]. In our study, social support, optimism, and coping based on positive attitude explained less stoicism in men. Stoic individuals who perceive less social support, are less optimistic, and display a passive style of coping with illness, tend to be more withdrawn — all related with worse adjustment to their disease. In contrast, Behen JM and Rodriguez JR found no correlation between resignation, social support, and coping with illness [27]. A review of eleven studies concluded that the experience of living with cancer in old age is characterized by profound ambiguity and a predominance of social withdrawal [28]. In women, stoicism was more common in older aged individuals [1] and was associated with less perceived social support and lower levels of optimism. In line with our series, Schou I et al. found a correlation between pessimism as quantified by the LOT-R scale, maladaptive coping, and depression in their study of women with breast cancer conducted one year following surgery [29].

This study has certain limitations. First, given its cross-sectional design, a causal interpretation cannot be made of the association between stoicism and psychological variables. Stoic perspective could only be assessed at a given point in time and not throughout the entire process of adapting to cancer. Secondly, we have evaluated patients' subjective appraisal of their social network without estimating the actual amount of support they received, or the support perceived by the family. Thirdly, stoicism and psychological traits were not evaluated prior to the diagnosis of cancer; as a result, the impact cancer had on them cannot be estimated.

In conclusion, stoicism was a factor that was associated with worse adaptation to cancer and was more common in males and in older participants. A stoic attitude correlated with perception of less social support, decreased optimism, passive coping strategies (helplessness and anxious preoccupation), and greater psychological distress (depression).

Declarations

Conflicts of interest

The authors have declared no conflict of interest related to this study.

Ethics approval

The study was approved by the Research Ethics Committee of the Principality of Asturias (19 January 2015) and by the Spanish Agency of Medicines and Medical Devices (AEMPS) (numberto: L34LM-MM2GH-Y925U-RJDHQ).

Research involving Human Participants

The study has been performed in accordance with the ethical standards of the 1964 Declaration of Helsinki and its later amendments. This study is an observational, non-interventionist trial.

Consent to participate and for publication

Signed informed consent was obtained from all patients.

Consent for publication

Informed consent and approval by the national competent authorities includes permission for publication and diffusion of the data.

Availability of data and material (data transparency)

The database is available through a centralized web platform: www.neocoping.es.

Code availability

Patients are identified by an encrypted code known only to the local researcher.

Acknowledgements

The authors would like to thank the investigators of the NEOcoping study, the Supportive Care Working Group of the Spanish Society of Medical Oncology (SEOM), Priscilla Chase Duran for editing the manuscript, and Natalia Cateriano, Miguel Vaquero, and IRICOM S.A. for the support of the website registry.

Authors' contributions

A.C.B., P.J.F. and C.C. developed the project, analyzed the data and drafted the manuscript. The other authors recruited patients and provided clinical information, comments, and improvements to the manuscript. All authors participated in the interpretation and discussion of data, and the critical review of the manuscript.

References

1. Moore A, Grime J, Campbell P, Richardson J (2013). Troubling stoicism: Sociocultural influences and applications to health and illness behaviour. Health 17:159–173.
2. Wagstaff GF, Rowledge AM (1995). Stoicism: Its relation to gender, attitudes toward poverty, and reactions to emotive material. J Soc Psychol 135:181–184.
3. Spiers J (2006). Expressing and responding to pain and stoicism in home ‐ care nurse–patient interactions. Scand J Caring Sci 20:293–301.
4. Garro LC (1990). Culture, pain and cancer. J Palliat Care 6:34-44.
5. Hillier R (1990). Control of pain in terminal cancer. Br Med Bull 46:279–291.
6. Hinton J (1994). Which patients with terminal cancer are admitted from home care? Palliat Med 8:197–210.
7. Calderon C, Ferrando PJ, Lorenzo-Seva U, Carmona-Bayonas A, Jara C, Ayala de la peña F, Beato C, Ramchandani A, García T, Mangas-Izquierdo M, Castelo B, Donnay O, Muñoz MM, Martínez de Castro E, García S, Ghanem I, Mellen T, Jiménez-Fonseca P (2017). Psychometric properties of Liverpool Stoicism Scale (LSS) in a cohort of patients with resected cancer in adjuvant treatment. Ann Psych. 2017;33:621.
8. Zimet GD, Powell SS, Farley GK, Werkman S, Berkoff KA (1990). Psychometric characteristics of the Multidimensional Scale of Perceived Social Support. J Pers Assess 55:610–617.
9. Vaillo YA, Pérez SM, López PM, Retes RR (2018). Mini-Mental Adjustment to Cancer Scale: Construct validation in Spanish breast cancer patients. J Psychosom Res 114:38–44.
10. Bredal IS (2010). The Norwegian version of the Mini ‐ Mental Adjustment to Cancer Scale: factor structure and psychometric properties. Psycho ‐ Oncol 19:216–221.
11. Galdón MJ, Durá E, Andreu Y, Ferrando M, Murgui S, Pérez S, Ibañez E (2008). Psychometric properties of the Brief Symptom Inventory-18 in a Spanish breast cancer sample. J Psychosom Res 65:533–539.
12. Cano-García FJ, Sanduvete-Chaves S, Chacón-Moscoso S, Rodríguez-Franco L, García-Martínez J, Antuña-Bellerín MA, Pérez-Gil JA (2015). Factor structure of the Spanish version of the Life Orientation Test-Revised (LOT-R): Testing several models. Int J Clin Heal Psychol 15:139–148.
13. Merckaert I, Libert Y, Messin S, Milani M, Slachmuylder J, Razavi D (2010). Cancer patients' desire for psychological support: prevalence and implications for screening patients' psychological needs. Psycho ‐ Oncol 19:141–9.

14. Veloso AG, Sperling C, Holm LV, Nicolaisen A, Rottmann N, Thayssen S, Christensen RD, Knudsen JL, Hansen DG (2013). Unmet needs in cancer rehabilitation during the early cancer trajectory–a nationwide patient survey. Acta Oncol 52:372–381.
15. Burg MA, Adorno G, Lopez EDS, Loerzel V, Stein K, Wallace C, Sharma DKB (2015). Current unmet needs of cancer survivors: Analysis of open‐ended responses to the American Cancer Society Study of Cancer Survivors II. Cancer 121:623–630.
16. Addis ME, Mahalik JR (2003). Men, masculinity, and the contexts of help seeking. Am Psychol 58:5.
17. Sobralske M (2006). Machismo sustains health and illness beliefs of Mexican American men. J Am Acad Nurse Pract 18:348–350.
18. Pettingale K, Morris T, Greer S, Haybittle JL (1985). Mental attitudes to cancer: an additional prognostic factor. Lancet 325:750.
19. Behen JM, Rodrigue JR (1994). Predictors of coping strategies among adults with cancer. Psychol Rep 74:43–48.
20. Hughes N, Closs SJ, Clark D (2009). Experiencing cancer in old age: a qualitative systematic review. Qual Health Res 19:1139–1153.
21. Schou I, Ekeberg Ø, Ruland CM, Sandvik L, Kåresen R (2004). Pessimism as a predictor of emotional morbidity one year following breast cancer surgery. Psycho‐Oncol 13:309–320.

TABLES (5)

Table 1. Patient characteristics (n =932)

Characteristic	N	%
Gender		
Female	566	60.7
Males	366	39.3
Age, years		
Less than 45	144	15.5
46 – 55	203	21.8
56 – 65	265	28.4
More than 66	320	34.3
Cancer type		
Colon	391	42.0
Breast	321	34.4
Stomach	42	4.5
Others	178	19.1
Cancer stage		
I	130	14.0
II	390	41.9
III	408	43.8
Missing	3	0.3
Educational level		
Less than high school	500	53.6
More than high school	432	46.4
Marital status		
Married/ partnered	710	76.3
Single	80	8.4
Separated/ divorced	79	8.5
Widowed	63	6.8
Employment status		
Full or part-time	540	58.0
Retired, homemaker, unemployed	392	42.0

Table 2. Gender differences in age, perception of risk of recurrence and toxicity, stoicism, and psychological scales in patients with cancer

	Men (n=366)		Women (n=566)		t-value	
**Variables**	**Mean**	**SD**	**Mean**	**SD**	**T**	**p**
Age (years)	62.1	12.1	56.9	11.8	6.549	0.001*
Perception of risk of:						
Recurrence with CT	78.6	22.1	78.1	21.6	0.320	---
Recurrence without CT	36.7	21.<br>4	32.4	22.5	2.711	0.007*
CT-related toxicity	53.7	22.7	59.4	22.4	-3.717	0.001*
LSS. Stoicism	58.4	7.2	54.2	7.7	8.388	0.001*
MSPSS. Social support	74.7	9.3	75.7	8.9	-1.634	---
Mini-MAC. Helplessness	19.8	19.1	19.2	19.5	0.536	---
Mini-MAC. Anxious preoccupation	36.7	21.6	42.2	23.6	-3.554	0.001*
Mini-MAC. Positive attitude	74.5	17.1	76.1	15.7	-1.404	---
Mini-MAC. Cognitive avoidance	51.5	26.3	54.8	26.0	-1.887	---
BSI-18. Somatization	59.7	6.7	61.8	6.9	-4.547	0.001*
BSI-18. Depression	59.2	6.7	61.5	6.0	-5.802	0.001*
BSI-18. Anxiety	59.7	7.0	63.0	6.9	-7.384	0.001*
LOT-R. Optimism	20.7	3.7	21.1	4.2	-1.478	---

Abbreviations: SD, Standard Deviation; CT, chemotherapy; LSS, Liverpool Stoicism Scale; MSPSS, Multidimensional Scale of Perceived Social Support; Mini-MAC, Mini-Mental Adjustment to Cancer; BSI, Brief Symptom Inventory; LOT-R, Life Orientation Test-Revised
* Statistically significant differences

Table 3. Comparison of cancer patients with high and low stoicism by age, perception of risk of recurrence and toxicity, and psychological scales

	Low stoicism (PC <25) n =250		High stoicism (PC >75) n =312		t-test for equality of means[a]	
**Variables**	Mean	SD	Mean	SD	t	P
Age (years)	64.4	12.1	61.4	11.6	-6.941	0.001*
Perception of risk of:						
Recurrence with CT	78.2	21.4	78.9	22.1	-0.412	---
Recurrence without CT	32.9	23.3	36.8	23.6	-1.932	---
Toxicity with CT	58.7	23.1	57.4	23.0	0.673	---
MSPSS. Social support	78.7	6.4	71.9	10.6	8.857	0.001*
Mini-MAC. Helplessness	13.8	16.9	20.8	19.9	-4.393	0.001*
Mini-MAC. Anxious preoccupation	39.1	22.6	42.4	24.5	-2.610	0.009*
Mini-MAC. Positive attitude	78.6	14.8	70.9	17.8	5.498	0.001*
Mini-MAC. Cognitive avoidance	51.6	26.8	52.3	26.1	-0.325	---
BSI-18. Somatization	61.6	6.9	61.3	7.2	0.487	---
BSI-18. Depression	60.2	5.5	61.6	6.5	-2.609	0.009*
BSI-18. Anxiety	62.1	7.7	62.3	8.3	-0.229	---
LOT-R. Optimism	22.1	4.2	20.0	3.9	5.990	0.001*

Abbreviation: PC, percentile, SD, Standard Deviation; CT, chemotherapy; LSS, Liverpool Stoicism Scale; MSPSS, Multidimensional Scale of Perceived Social Support; Mini-MAC, Mini-Mental Adjustment to Cancer; BSI, Brief Symptom Inventory; LOT-R, Life Orientation Test-Revised

[a] Before t-test analysis, Levene's test for equality of variances was applied.

* Statistically significant differences

Table 4. Correlation between stoicism, perception of risk of recurrence and toxicity, psychological scales in men and women with cancer

	Men		Women	
**Variables****	***r***	***p***	***r***	***p***
Age (years)	0.044	0.398	0.261	0.001*
Perception of risk of:				
Recurrence with CT	-0.031	---	0.020	---
Recurrence without CT	0.139	0.008*	-0.050	---
Toxicity with CT	-0.051	---	-0.030	---
MSPSS. Social support	-0.304	0.001*	-0.286	0.001*
Mini-MAC. Helplessness	0.130	0.013*	0.189	0.001*
Mini-MAC. Anxious preoccupation	0.199	0.001*	0.101	0.018*
Mini-MAC. Positive attitude	-0.198	0.001*	-0.149	0.038*
Mini-MAC. Cognitive avoidance	0.018	---	0.079	---
BSI-18. Somatization	0.041	---	0.012	---
BSI-18. Depression	0.176	0.001*	0.144	0.001*
BSI-18. Anxiety	0.133	0.011*	0.052	---
LOT-R. Optimism	-0.276	0.001*	-0.257	0.001*

Abbreviations: SD, Standard Deviation; LSS, Liverpool Stoicism Scale; MSPSS, Multidimensional Scale of Perceived Social Support; Mini-MAC, Mini-Mental Adjustment to Cancer; BSI, Brief Symptom Inventory; LOT-R, Life Orientation Test-Revised

* Statistically significant differences

**Criterion variable: stoicism according to LSS scale

Table 5. Summary of the linear regression analysis predicting for stoicism; corrected R (R^2), non-standardized coefficient (B), standard error, and standardized beta coefficient (β); analysis of variance and significance level

							95% IC	
**Predictor**	**R^2**	**B**	**SE**	**β**	**t**	***p***	**Lower**	**Upper**
**Men**								
(Constant)		78.171	3.308		23.634	0.001	71.666	84.676
Social Support[1]	15.6	-0.192	0.040	-0.246	-4.833	0.001	-0.270	-0.114
Optimism[2]		-0.348	0.101	-0.180	-3.440	0.001	-0.546	-0.149
Anxious preoccupation[3]		0.049	0.017	-0.145	2.886	0.004	0.015	0.082
**Women**								
(Constant)		67.399	3.272		20.600	0.001	60.972	73.826
Social support[1]	16.9	-0.189	0.035	-0.218	-5.273	0.001	-0.256	-0.117
Age		0.145	0.026	0.223	5.584	0.001	0.094	0.196
Optimism[2]		-0.349	0.075	-0.190	-4.634	0.001	-0.497	-0.201

Abbreviations: R^2, corrected R; B, non-standardized coefficient; SE, standard error; β, standard beta coefficient; t, analysis of variance; p, significance level, IC, interval confidence; LSS, Liverpool Stoicism Scale; MSPSS, Multidimensional Scale of Perceived Social Support; Mini-MAC, Mini-Mental Adjustment to Cancer; LOT-R, Life Orientation Test-Revised

[1]MSPSS. [2]LOT-R. [3]Mini-MAC subscale.

Criterion variable: stoicism according to LSS

Dear Editor-in-Chief,

In accordance with the Editor's suggestions and concerns regarding the manuscript entitled, "Stoic attitude in patients with cancer from the NEOcoping study: cross-sectional study", please find the new version enclosed.

On behalf of all the co-authors, I would like to thank the editor and the reviewers for their thoughtful and insightful comments on our work, which we have considered very closely, while preparing this revised version of the manuscript as detailed in the following point-by-point reply.

The new text has been added in blue font in the revised version of the article.

We trust that we have addressed all the issues raised by the reviewers to their satisfaction and you now find the manuscript suitable for publication in your journal.

Thank-you very much for your consideration.

Respectfully, Dra Caterina Calderon

Reviewer #1: This manuscript explored the association of stoicism with coping, psychological distress, optimism, perceived social support, and estimated risk of recurrence in cancer patients. The present study has several strengths: a well-written manuscript, clinical relevance of the study objective to the cancer survivorship and a relatively large sample size. Nevertheless, the reviewer has the following comments and criticism on the conceptual framework and methodology of the study, particularly on the incremental value of stoicism over existing cancer coping strategies.

Well aware of the value your critical reading brings to our text, we would like to begin by thanking the reviewer for having read the text and for considering that it may be of scientific interest and for having captured the highlights and strengths of this work. Please find below our response to each of the comments made by the reviewer.

1) Introduction: Stoicism is a interesting concept that has rarely been examined in the context of coping with cancer. The authors brought up this innovative construct and could potentially contribute to the existing literature. However, the current introduction is too brief and much more information is needed to establish the importance of the concept of stoicism in the context of cancer. The first two paragraphs are just overall introduction of stoicism without specific relevance to cancer. The authors need to provide a more focused background of the study while adding more citations from relevant literature. There are currently only 6 references in the Introduction!

In response to this question, we have expanded the explanation of the relevance and implication of stoicism in cancer patients and the available scientific evidence on this issue.

To clarify this point, the following paragraph has been added to the **Introduction section**:

"A study of men with prostate cancer of any stage suggests that stoicism is neither adaptive nor maladaptive for distress and quality of life outcomes and is not highly correlated with male self-esteem or psychological flexibility. The authors hypothesized that this may mean that men use stoicism to cope with prostate cancer in line with their own masculine values. In another study by Mah et al., younger, and older patients with advanced cancer showed unique biopsychosocial correlates of pain-related stoicism and caution** Cognitive avoidance of distressing thoughts was intrapersonal, associated with greater concealment, self-doubt, and reticence in the elderly. Older adults were more likely to regulate emotions, to be less expressive about negative events and avoidance of negative information in relation to a more stoic attitude."*

*McAteer G, Gillanders D. Investigating the role of psychological flexibility, masculine self-esteem and stoicism as predictors of psychological distress and quality of life in men living with prostate cancer. Eur J Cancer Care (Engl). 2019;28(4):e13097..

**Mah K, Tran KT, Gauthier LR, et. Do Correlates of Pain-Related Stoicism and Cautiousness Differ in Younger and Older People With Advanced Cancer? J Pain. 2018;19(3):301-316.

2) What does the last sentence in Introduction "Finally, we have developed a model of stoicism correlating with coping, psychological stress, optimism, and perceived social support" mean? Is it one of the study objectives?

The final sentence of the introduction does not correspond to either of the two objectives mentioned above and has therefore been deleted.

3) Given the **cross-sectional nature of this study**, implications of the current results are limited and measurement of the estimated risk of future recurrence might not be appropriate and relevant.

As the reviewer refers, cross-sectional nature of this study is a limiting factor and this is stated in the first limitation in the **discussion section**: "*First, given its cross-sectional design, a causal interpretation cannot be made of the association between stoicism and psychological variables. Stoic perspective could only be assessed at a given point in time and not throughout the entire process of adapting to cancer.*"

Patients were asked about their perceived risk of recurrence before starting chemotherapy adjuvant/complementary to surgery with curative intent. Since tumor stage was similar, the influence of stoicism, gender and other psychological variables on patients' perception of their risk of cancer recurrence was assessed.

Therefore, the patient's perception of their risk of cancer recurrence was considered, but no calculation of the risk of recurrence was made based on any of the variables studied. Therefore, the analyses do not establish causal relationships in oncological outcomes.

4) **Mini-MAC**: In page 4, the authors wrote that the Mini-MAC assesses **five cognitive coping responses**, but they only listed helplessness, anxious preoccupation, positive attitude, and cognitive avoidance as the **4 factors**. It seems the authors have somehow adopted the 4-factor structure for Mini-MAC advocated in the Norwegian version they cited in [10]. However, as the authors have quoted, the Mini-MAC is generally perceived to have 5 factors with "positive attitude" being distinguished into fighting spirit (4 items) and 5) fatalism (5 items). The authors need to better a stronger justification not to opt for the conventional factor structure for the Mini-MAC given it is a well-established and validated scale of cancer coping.

The authors of this paper validated the Spanish version of the Mini-MAC in a Spanish series of cancer patients*.

The results of the factor analysis indicated a 4-factor structure. Three subscales have similar psychometric properties to the Helplessness, Anxious preoccupation, and Cognitive avoidance subscales of the original Mini-MAC and the Fighting Spirit and Fatalism subscales were combined into the Positive Attitude scale. The scale scores derived from the four factors showed acceptable accuracy for individual measures, as well as stability across test-retest assessments at 6 months. Validity assessments found meaningful relations between the derived scale scores, and Brief Symptom Inventory (BSI) depression and anxiety scores and Functional Assessment of Chronic Illness Therapy (FACIT) spiritual well-being scores.

Therefore, given that our team had validated and confirmed in previous work the accuracy of a 4-factor based scale, it was decided to use it in this work.

In the method section, we have added the following clarification regarding this point:

"The 29-item mini-MAC assesses five cognitive coping responses: helplessness, anxious preoccupation, fighting spirit, fatalism and cognitive avoidance. For this study, the version with a 4-factor structure validated in Spanish was used, grouping fighting spirit and fatalism into a single factor, positive attitude."*

*Calderon C, Lorenzo-Seva U, Ferrando PJ, et al. Psychometric properties of Spanish version of the Mini-Mental Adjustment to Cancer Scale. Int J Clin Health Psychol. 2021;21(1):100185. doi: 10.1016/j.ijchp.2020.06.001.

5) **Incremental value of stoicism**: Following the last comment on Mini-MAC, the authors need to delineate the conceptual distinction between stoicism and existing cancer coping strategies such as fatalism and cognitive avoidance as Mini-MAC factors. It seems to the reviewer that stoicism has conceptual overlap with both fatalism and cognitive avoidance. It is essential to specify the conceptual difference between stoicism and existing cancer coping strategies to establish the need for the present study.

The term fatalism refers to the belief in the determinism of events, directed by causes independent of human will. This notion of fatalism carries a negative connotation. While Stoicism is based on the notion of control over life-disturbing emotions, the goal is based on tolerance and self-control. We have made changes to the introduction to delineate the concepts.

6) Analysis: The authors conduct tests on gender and age differences in stoicism and other psychological variables. Did they consider ANOVAs to test the potential interaction effects between age and gender? This should be feasible given the large sample size. The findings presented in Tables 2 – 4 are mostly **t-tests and correlational analysis**, which are rather basic and do not take full account of the complex relationships among the variables.

You are right, it would be interesting to analyze the interaction between stoicism and sex and age and we will take that into account for future studies. The objective of the present study was to analyze the differences between patients with high and low levels of stoicism.

7) Why did the **authors dichotomize the continuous stoicism score into high and low stoicism subgroups**? What is the added value of conducting t-tests across the high-low subgroups over correlation as in Table 4? The results in Tables 3 and 4 appear to be redundant without new information added.

To carry out the statistical analyzes presented in tables 2 and 4, we rely on the scientific literature that indicates that significant differences are observed between men and women in stoicism. The correlation of stoicism with the psychological variables according to sex was carried out first, in order to know which of these would be predictive in explaining stoicism (Table 5), also differentiating it by sex.

8) The authors conducted the correlation analyses in **male and female subsamples** separately in Table 4. Are the gender differences significant? Most of the correlations in Table 3 are rather small and < 0.30. Do they bear much clinical relevance to the participants?

To carry out the statistical analyzes presented in tables 2 and 4, we rely on the scientific literature that indicates that significant differences are observed between men and women in stoicism. The correlation of stoicism with the psychological variables according to sex was carried out first, in order to know which of these would be predictive in explaining stoicism (Table 5), also differentiating it by sex.

9) Most of the variables in the study are **coping strategies (social support, Mini-MAC, optimism).** Why did the authors use these variables to predict stoicism in Table 5? Is stoicism perceived as a predictor of cancer symptoms/distress or outcome of other coping strategies in this study? Even though this study is **only cross-sectional** in nature and could not answer the causal direction

among the study variables, it would be better if the authors could provide a clearer conceptual framework of the study variables related to the main construct of stoicism.

Our study aims to explain the percentage of explained variance of various psychological variables in stoicism, it does not try to explain how stoicism develops, for this we would need to carry out a longitudinal study. Following your recommendations, we have improved the conceptual framework of the introduction.

10) The authors wrote they conducted structural equation models for the regression analyses. However, a lot of information is missing for the SEM analysis, such as model specification, model fit, model modification, control variables in the model. How can they conduct SEM in SPSS 23.0 and have F-values in the results in SEM analysis? Presentation of the last part of results is too brief for the reviewer and readers to understand. Did the authors use latent factors of the predictors or outcomes in the SEM analysis?

As described in Data Analysis, in the manuscript we use two linear regression analyzes to predict the association between stoicism and psychological scales in men and women. In subsequent statistical analyses, stoicism served as the criterion variable in regression analyses, while social support (according to the MSPSS), coping strategies (Mini-MAC), psychological distress (BSI-18) and optimism (LOT-R) were used as predictors.
We have not used SEM. We are sorry.

Reviewer #2: In the article there is no theoretical background of concepts measured in the study, except stoic attitude. The other variables included in the research should be also described in the introduction.

In response to the reviewer's request, we have added the following paragraph in the introduction section relating to the other items, coping, psychological distress, optimism, and perception of social support:

"As cancer is a stressful event associated with fear and prognostic uncertainty, it is common for patients to employ coping strategies to deal with it. Stoicism and emotional distress (anxiety, depression, and somatization) may interfere with coping by increasing anxious preoccupation. Dispositional optimism may act as a resource that maintains a positive mood, provides a more flexible coping style to face with the inability to control stressful stimuli, and protects patients from the possible negative effects of cancer treatment and cancer itself in case of recurrence**,***. Likewise, social support, defined as the perception that others are willing to help, appears to operate similarly to optimism and is a critical factor in decreasing negative psychological affect in individuals with cancer****."*

*Jimenez-Fonseca P, Calderón C, Hernández R, et al. Factors associated with anxiety and depression in cancer patients prior to initiating adjuvant therapy. Clin Transl Oncol. 2018;20(11):1408-1415. doi: 10.1007/s12094-018-1873-9.

**Ciria-Suarez L, Calderon C, Fernández Montes A, et al. Optimism and social support as contributing factors to spirituality in Cancer patients. Support Care Cancer. 2021;29(6):3367-3373. doi: 10.1007/s00520-020-05954-4.

***Ciria-Suarez L, Jiménez-Fonseca P, Palacín-Lois M, et al. Breast cancer patient experiences through a journey map: A qualitative study. PLoS One. 2021 Sep 22;16(9):e0257680. doi: 10.1371/journal.pone.0257680.

****Calderon C, Gomez D, Carmona-Bayonas A, et al. Social support, coping strategies and sociodemographic factors in women with breast cancer. Clin Transl Oncol. 2021;23(9):1955-1960. doi: 10.1007/s12094-021-02592-y

In the methodological part, we have little information about specific cancer diagnosis. Cancer patients have different e. g. coping styles or depressive symptoms regarding the specific diagnosis. For example, patients may differ in depression level regarding to the diagnosis. It should be explained, why Authors of the manuscript did not expect and measure any differences in stoic attitude among patient with different diagnosis.

Analyzing the differences in depression and coping strategies among cancer patients is very interesting, but it is not the objective of our study.

In the article there are some grammatical mistakes which need to be corrected, such as: p. 5 "Gender and stoicism differences on age, perception of life expectancy, and risk of toxicity, and psychological scales were measures using independent samples t-tests." - should be ..."were measured". p. 8: "In our study, social support, optimism, and coping based on positive attitude explined less stoicism in men." - should be: "... explained...".

Following the reviewer's indications, we have checked the text in detail and corrected the two spelling mistakes and one other error that we have detected.

We have highlighted them in blue in the text.

Cognizant of the contribution their insightful and critical observations have made to enhancing the quality of the text, the researchers of this study would again like to thank the Editor and reviewers for their time and effort.
