# Supplementary material for: Stoic attitude in patients with cancer from the NEOcoping study: Cross-sectional study
Source: PLoS One. 2022 Jul 28;17(7):e0269712. doi: 10.1371/journal.pone.0269712 (PMC9333303; doi:10.1371/journal.pone.0269712)
Supplement: Attachment — Submitted filename: Stoicism Response to Reviewers FINAL.docx [file pone.0269712.s002.docx]

Dear Editor-in-Chief,

In accordance with the Editor’s suggestions and concerns regarding the manuscript entitled, “Stoic attitude in patients with cancer from the NEOcoping study: cross-sectional study”, please find the new version enclosed.

On behalf of all the co-authors, I would like to thank the editor and the reviewers for their thoughtful and insightful comments on our work, which we have considered very closely, while preparing this revised version of the manuscript as detailed in the following point-by-point reply.

The new text has been added in blue font in the revised version of the article.

We trust that we have addressed all the issues raised by the reviewers to their satisfaction and you now find the manuscript suitable for publication in your journal.

Thank-you very much for your consideration.

Respectfully, Dra Caterina Calderon

**Reviewer #1:** This manuscript explored the association of stoicism with coping, psychological distress, optimism, perceived social support, and estimated risk of recurrence in cancer patients. The present study has several strengths: a well-written manuscript, clinical relevance of the study objective to the cancer survivorship and a relatively large sample size. Nevertheless, the reviewer has the following comments and criticism on the conceptual framework and methodology of the study, particularly on the incremental value of stoicism over existing cancer coping strategies.

Well aware of the value your critical reading brings to our text, we would like to begin by thanking the reviewer for having read the text and for considering that it may be of scientific interest and for having captured the highlights and strengths of this work. Please find below our response to each of the comments made by the reviewer.

1) Introduction: Stoicism is a interesting concept that has rarely been examined in the context of coping with cancer. The authors brought up this innovative construct and could potentially contribute to the existing literature. However, the current introduction is too brief and much more information is needed to establish the importance of the concept of stoicism in the context of cancer. The first two paragraphs are just overall introduction of stoicism without specific relevance to cancer. The authors need to provide a more focused background of the study while adding more citations from relevant literature. There are currently only 6 references in the Introduction!

In response to this question, we have expanded the explanation of the relevance and implication of stoicism in cancer patients and the available scientific evidence on this issue.

To clarify this point, the following paragraph has been added to the **Introduction section**:

*“A study of men with prostate cancer of any stage suggests that stoicism is neither adaptive nor maladaptive for distress and quality of life outcomes and is not highly correlated with male self-esteem or psychological flexibility. The authors hypothesized that this may mean that men use stoicism to cope with prostate cancer in line with their own masculine values*. In another study by Mah et al., younger, and older patients with advanced cancer showed unique biopsychosocial correlates of pain-related stoicism and caution** Cognitive avoidance of distressing thoughts was intrapersonal, associated with greater concealment, self-doubt, and reticence in the elderly. Older adults were more likely to regulate emotions, to be less expressive about negative events and avoidance of negative information in relation to a more stoic attitude.”*

*McAteer G, Gillanders D. Investigating the role of psychological flexibility, masculine self-esteem and stoicism as predictors of psychological distress and quality of life in men living with prostate cancer. Eur J Cancer Care (Engl). 2019;28(4):e13097..

**Mah K, Tran KT, Gauthier LR, et. Do Correlates of Pain-Related Stoicism and Cautiousness Differ in Younger and Older People With Advanced Cancer? J Pain. 2018;19(3):301-316.

2) What does the last sentence in Introduction “Finally, we have developed a model of stoicism correlating with coping, psychological stress, optimism, and perceived social support” mean? Is it one of the study objectives?

The final sentence of the introduction does not correspond to either of the two objectives mentioned above and has therefore been deleted.

3) Given the **cross-sectional nature of this study**, implications of the current results are limited and measurement of the estimated risk of future recurrence might not be appropriate and relevant.

As the reviewer refers, cross-sectional nature of this study is a limiting factor and this is stated in the first limitation in the **discussion section**: "*First, given its cross-sectional design, a causal interpretation cannot be made of the association between stoicism and psychological variables. Stoic perspective could only be assessed at a given point in time and not throughout the entire process of adapting to cancer.*”

Patients were asked about their perceived risk of recurrence before starting chemotherapy adjuvant/complementary to surgery with curative intent. Since tumor stage was similar, the influence of stoicism, gender and other psychological variables on patients' perception of their risk of cancer recurrence was assessed.

Therefore, the patient's perception of their risk of cancer recurrence was considered, but no calculation of the risk of recurrence was made based on any of the variables studied. Therefore, the analyses do not establish causal relationships in oncological outcomes.

4) **Mini-MAC**: In page 4, the authors wrote that the Mini-MAC assesses **five cognitive coping responses**, but they only listed helplessness, anxious preoccupation, positive attitude, and cognitive avoidance as the **4 factors**. It seems the authors have somehow adopted the 4-factor structure for Mini-MAC advocated in the Norwegian version they cited in [10]. However, as the authors have quoted, the Mini-MAC is generally perceived to have 5 factors with “positive attitude” being distinguished into fighting spirit (4 items) and 5) fatalism (5 items). The authors need to better a stronger justification not to opt for the conventional factor structure for the Mini-MAC given it is a well-established and validated scale of cancer coping.

The authors of this paper validated the Spanish version of the Mini-MAC in a Spanish series of cancer patients*.

The results of the factor analysis indicated a 4-factor structure. Three subscales have similar psychometric properties to the Helplessness, Anxious preoccupation, and Cognitive avoidance subscales of the original Mini-MAC and the Fighting Spirit and Fatalism subscales were combined into the Positive Attitude scale. The scale scores derived from the four factors showed acceptable accuracy for individual measures, as well as stability across test-retest assessments at 6 months. Validity assessments found meaningful relations between the derived scale scores, and Brief Symptom Inventory (BSI) depression and anxiety scores and Functional Assessment of Chronic Illness Therapy (FACIT) spiritual well-being scores.

Therefore, given that our team had validated and confirmed in previous work the accuracy of a 4-factor based scale, it was decided to use it in this work.

In the method section, we have added the following clarification regarding this point:

*“The 29-item mini-MAC assesses five cognitive coping responses: helplessness, anxious preoccupation, fighting spirit, fatalism and cognitive avoidance. For this study, the version with a 4-factor structure validated in Spanish was used, grouping fighting spirit and fatalism into a single factor, positive attitude*.”*

*Calderon C, Lorenzo-Seva U, Ferrando PJ, et al. Psychometric properties of Spanish version of the Mini-Mental Adjustment to Cancer Scale. Int J Clin Health Psychol. 2021;21(1):100185. doi: 10.1016/j.ijchp.2020.06.001.

5) **Incremental value of stoicism**: Following the last comment on Mini-MAC, the authors need to delineate the conceptual distinction between stoicism and existing cancer coping strategies such as fatalism and cognitive avoidance as Mini-MAC factors. It seems to the reviewer that stoicism has conceptual overlap with both fatalism and cognitive avoidance. It is essential to specify the conceptual difference between stoicism and existing cancer coping strategies to establish the need for the present study.

The term fatalism refers to the belief in the determinism of events, directed by causes independent of human will. This notion of fatalism carries a negative connotation. While Stoicism is based on the notion of control over life-disturbing emotions, the goal is based on tolerance and self-control. We have made changes to the introduction to delineate the concepts.

6) Analysis: The authors conduct tests on gender and age differences in stoicism and other psychological variables. Did they consider ANOVAs to test the potential interaction effects between age and gender? This should be feasible given the large sample size. The findings presented in Tables 2 – 4 are mostly **t-tests and correlational analysis**, which are rather basic and do not take full account of the complex relationships among the variables.

You are right, it would be interesting to analyze the interaction between stoicism and sex and age and we will take that into account for future studies. The objective of the present study was to analyze the differences between patients with high and low levels of stoicism.

7) Why did the **authors dichotomize the continuous stoicism score into high and low stoicism subgroups**? What is the added value of conducting t-tests across the high-low subgroups over correlation as in Table 4? The results in Tables 3 and 4 appear to be redundant without new information added.

To carry out the statistical analyzes presented in tables 2 and 4, we rely on the scientific literature that indicates that significant differences are observed between men and women in stoicism. The correlation of stoicism with the psychological variables according to sex was carried out first, in order to know which of these would be predictive in explaining stoicism (Table 5), also differentiating it by sex.

8) The authors conducted the correlation analyses in **male and female subsamples** separately in Table 4. Are the gender differences significant? Most of the correlations in Table 3 are rather small and < 0.30. Do they bear much clinical relevance to the participants?

To carry out the statistical analyzes presented in tables 2 and 4, we rely on the scientific literature that indicates that significant differences are observed between men and women in stoicism. The correlation of stoicism with the psychological variables according to sex was carried out first, in order to know which of these would be predictive in explaining stoicism (Table 5), also differentiating it by sex.

9) Most of the variables in the study are **coping strategies (social support, Mini-MAC, optimism).** Why did the authors use these variables to predict stoicism in Table 5? Is stoicism perceived as a predictor of cancer symptoms/distress or outcome of other coping strategies in this study? Even though this study is **only cross-sectional** in nature and could not answer the causal direction among the study variables, it would be better if the authors could provide a clearer conceptual framework of the study variables related to the main construct of stoicism.

Our study aims to explain the percentage of explained variance of various psychological variables in stoicism, it does not try to explain how stoicism develops, for this we would need to carry out a longitudinal study. Following your recommendations, we have improved the conceptual framework of the introduction.

10) The authors wrote they conducted structural equation models for the regression analyses. However, a lot of information is missing for the SEM analysis, such as model specification, model fit, model modification, control variables in the model. How can they conduct SEM in SPSS 23.0 and have F-values in the results in SEM analysis? Presentation of the last part of results is too brief for the reviewer and readers to understand. Did the authors use latent factors of the predictors or outcomes in the SEM analysis?

As described in Data Analysis, in the manuscript we use two linear regression analyzes to predict the association between stoicism and psychological scales in men and women. In subsequent statistical analyses, stoicism served as the criterion variable in regression analyses, while social support (according to the MSPSS), coping strategies (Mini-MAC), psychological distress (BSI-18) and optimism (LOT-R) were used as predictors.

We have not used SEM. We are sorry.

**Reviewer #2:** In the article there is no theoretical background of concepts measured in the study, except stoic attitude. The other variables included in the research should be also described in the introduction.

In response to the reviewer's request, we have added the following paragraph in the introduction section relating to the other items, coping, psychological distress, optimism, and perception of social support:

*“As cancer is a stressful event associated with fear and prognostic uncertainty, it is common for patients to employ coping strategies to deal with it. Stoicism and emotional distress (anxiety, depression, and somatization) may interfere with coping by increasing anxious preoccupation*. Dispositional optimism may act as a resource that maintains a positive mood, provides a more flexible coping style to face with the inability to control stressful stimuli, and protects patients from the possible negative effects of cancer treatment and cancer itself in case of recurrence**,***. Likewise, social support, defined as the perception that others are willing to help, appears to operate similarly to optimism and is a critical factor in decreasing negative psychological affect in individuals with cancer****.”*

*Jimenez-Fonseca P, Calderón C, Hernández R, et al. Factors associated with anxiety and depression in cancer patients prior to initiating adjuvant therapy. Clin Transl Oncol. 2018;20(11):1408-1415. doi: 10.1007/s12094-018-1873-9.

**Ciria-Suarez L, Calderon C, Fernández Montes A, et al. Optimism and social support as contributing factors to spirituality in Cancer patients. Support Care Cancer. 2021;29(6):3367-3373. doi: 10.1007/s00520-020-05954-4.

***Ciria-Suarez L, Jiménez-Fonseca P, Palacín-Lois M, et al. Breast cancer patient experiences through a journey map: A qualitative study. PLoS One. 2021 Sep 22;16(9):e0257680. doi: 10.1371/journal.pone.0257680.

****Calderon C, Gomez D, Carmona-Bayonas A, et al. Social support, coping strategies and sociodemographic factors in women with breast cancer. Clin Transl Oncol. 2021;23(9):1955-1960. doi: 10.1007/s12094-021-02592-y

In the methodological part, we have little information about specific cancer diagnosis. Cancer patients have different e. g. coping styles or depressive symptoms regarding the specific diagnosis. For example, patients may differ in depression level regarding to the diagnosis. It should be explained, why Authors of the manuscript did not expect and measure any differences in stoic attitude among patient with different diagnosis.

Analyzing the differences in depression and coping strategies among cancer patients is very interesting, but it is not the objective of our study.

In the article there are some grammatical mistakes which need to be corrected, such as: p. 5 "Gender and stoicism differences on age, perception of life expectancy, and risk of toxicity, and psychological scales were measures using
independent samples t-tests." - should be ..."were measured". p. 8: "In our study, social support, optimism, and coping based on positive attitude explined less stoicism in men." - should be: "... explained...".

Following the reviewer's indications, we have checked the text in detail and corrected the two spelling mistakes and one other error that we have detected.

We have highlighted them in blue in the text.

Cognizant of the contribution their insightful and critical observations have made to enhancing the quality of the text, the researchers of this study would again like to thank the Editor and reviewers for their time and effort.
