# Supplementary material for: Stoic attitude in patients with cancer from the NEOcoping study: Cross-sectional study
Source: PLoS One. 2022 Jul 28;17(7):e0269712. doi: 10.1371/journal.pone.0269712 (PMC9333303; doi:10.1371/journal.pone.0269712)
Supplement: Attachment — Submitted filename: 20200501 Response to reviwer STOICISM article.docx [file pone.0269712.s004.docx]

**PONE-D-20-38896R1**

**Stoic attitude in patients with cancer from the NEOcoping study: cross-sectional study**

**PLOS ONE**

Dear Dr. Calderón,

Thank you for submitting your manuscript to PLOS ONE. After careful consideration, we feel that it has merit but does not fully meet PLOS ONE’s publication criteria as it currently stands. Therefore, we invite you to submit a revised version of the manuscript that addresses the points raised during the review process.

The authors have in general conducted a satisfactory revision on the manuscript in view of the previous reviewer comments. However, the manuscript still needs to address the methodological concerns raised by Reviewer 3 regarding statistical analysis. As Reviewer 3 pointed out, it is necessary to carry out and include some additional statistical methods of greater capacity to predict more complex relational maps.

Please submit your revised manuscript by May 02 2022 11:59PM.

Please include the following items when submitting your revised manuscript:

- A rebuttal letter that responds to each point raised by the academic editor and reviewer(s). File labeled 'Response to Reviewers'.
- A marked-up copy of your manuscript that highlights changes made to the original version. File labeled 'Revised Manuscript with Track Changes'.
- An unmarked version of your revised paper without tracked changes. File labeled 'Manuscript'.

We look forward to receiving your revised manuscript.

Kind regards,

Ted C.T. Fong
Academic Editor
PLOS ONE

**Dear Editor-in-Chief,**

On behalf of all the co-authors, I would like to thank the editor and the reviewers for their thoughtful and insightful comments on our work, which we have considered very closely, while preparing this revised version of the manuscript as detailed in the following point-by-point reply.

The new text has been added in red font in the revised version of the article.

As the Editor indicates and following especially the recommendations of reviewer 3, we have adapted the stoicism theoretical model of Furnham et al., (2003), and Spiers, (2006). This stoicism model represents the relationship between several key constructs such as stoicism, social support, optimism, and coping strategies. In addition, we have conducted further statistical analyses (using SEM) to predict a complex model.

We trust that we have addressed all the issues raised by the reviewers to their satisfaction and you now find the manuscript suitable for publication in your journal.

Thank-you very much for your consideration.

Respectfully,

Dr Caterina Calderon

**Reviewers' comments:**

**Reviewer #2**: The article has been improved but there are still some issues to explain.

The Authors still did not refer to the question: "...why Authors of the manuscript did not expect and measure any differences in stoic attitude among patients with different diagnosis? (diagnosis of type of cancer of course).

**Comment**: Following your suggestion, we have performed the calculation and the results indicate that there are differences depending on the primary tumor (F= 10.445, p=0.001). Patients with colon cancer score higher on stoicism than patients with breast cancer (M: 57.4 vs M 53.8, IJ: 3.544, p=0.001, respectively).

-Colon (M: 57.4, SD: 7.3)

- Breast (M: 53.8, SD: 7.9)

- Stomach (M: 56.8; SD: 7.1)

- Others (M: 55.9; SD: 7.8)

We have included these in the manuscript (result section):

*“In terms of primary cancer, patients with colon cancer scored higher on stoicism than patients with breast cancer (M: 57.4 vs M 53.8; F= 10.445, p=0.001; IJ: 3.544, p=0.001, respectively).”*

There are still many linguistic errors and the style should be improved (e.g. in the sentense: "Stoicism has been associated with being male, and Wagstaff and Rowledge found that men exhibited higher stoicism scores; nevertheless, no studies on the associations with stoicism specifically in men and women with cancer").

**Comment**: The text was translated by a professional translator who has revised it again after the response to the queries, making modifications. In particular, this sentence has been removed from the text. The authors hope that this new version of the article will be better written for the reviewer.

**Reviewer #3:** The manuscript addresses a topic that is consistent with the scope and aims of this Journal. The purpose of the manuscript is important for health: the aim was to explore associations of stoicism with other outcomes in patients with non-metastatic cancer, and assess gender differences in age, perception of the risk of cancer relapse, the risk of adjuvant chemotherapy-related toxicity, coping, psychological stress, optimism, and perceived social support.

The **statistical analyses performed are adequate but too simple** (frequency, correlation, comparison of means, regression) to generate a novel impact or significant conclusions from the research question posed. It would be necessary to consider a greater impact of the data from analyses that allow more predictive conclusions. They informed that the margin error of the sample size was calculated (Cohen’s statistics). **The effect size has to be indicated as a relevant information in comparison of means in order to be able to properly interpret the statistical power of the result found.**

**Comment**:

We thank the reviewer for this suggestion, which has helped to increase the rigor and precision of the results.

Following this suggestion, we have included information on how to interpret the statistical power of the analyses performed and on the use of a Structural Equation Modeling (SEM) to estimate the correlation between variables. This is the text added in the methods section:

*“Effect sizes were calculated by Cohen’s statistics. Cohen’s d was reported as an indicator of the effect size of the differences, with d > 0.2 representing a small, d > 0.5 a medium and d > 0.8 a large effect size [27]. Bivariate correlations were calculated between stoicism (LSS) and psychological scales (MSPSS, Mini-Mac, BSI-18, and LOT-R) and sociodemographic factors. All data were checked for normality, outliers, and the assumptions of multicollinearity and homoscedasticity. Structural Equation Modeling (SEM) is a method for building, estimating, and testing theoretical models of the relationships between variables. It can be used instead of multiple regression and other methods to analyze the strength of correlations between individual variable indicators in a specific population [28]. In this study, the previously determined significant factors were used in the SEM to identify the relationship between stoicism and psychological scales. Standardized direct, indirect, and total effects with corresponding 95% bias-corrected confidence intervals (CI) were measure using the bootstrapping methods [28,29]. The model fit was tested using the normed χ2 value (NC; desired value < 2.0, desired significance P > 0.05), the goodness-of-fit index; the Comparative Fit Index (CFI), the Tucker-Lewis index (TLI), the Normed Fit index (NFI) (>0.95 indicating an excellent* fit); root mean square of approximation (RMSEA; desired value< 0.06) [28].”

Statistical analyses based on regression are adequate for prediction but trying a Path analysis, SEM or QCA analysis could improve the prediction capacity with such a big sample and could answer the predicting objectives of this study in a more complete way. A structural equation model (SEM) is needed for these prediction objectives and this big size of sample. Particularly, the structural equation model, (SEM) the estimate provided by the robust method of maximum likelihood estimation (ML), recommended to correct the possible absence of multivariate normality, is recommended to be applied in all cases. A structural equation model could be done to predict dependent variables through independent variables. In addition, multigroup have to be conducted to test the moderating effect of variables such as gender, type of cancer, severity of the disease (measured by variables such as prognosis, cancer stage, risk of cancer recurrence, toxicity). For example, in consideration of the dropout rate according to the criteria of Bae (2017) at least 200 participants required in path analysis with 12 or fewer observation. In this study we have a sample over 900 participants.

The QCA allows the quantitative analysis even in cases of a small number of participants (that is not particularly the case of this study) but using Boolean algebra as a formal tool to identify which of a series of factors (independent variables or causal conditions) are associated with the presence of a given result (criterion variable or result condition). Thus, it allows proposing pathways (which combine a particular interaction between the variables) to optimize the prediction of the independent variable. **It is necessary to carry out and include some additional statistical method of greater capacity to predict more complex relational maps.**

**Comment**: As suggested, we have adapted the stoicism theoretical model of Furnham et al., 2003; Spiers, 2006, described in the introduction. This stoicism model represents the relationship between several key constructs among stoicism, social support, optimism, and coping strategies.

We presume that: a) social support and optimism will positively affect patients' stoicism and coping strategies; b) stoicism will be related to coping strategies; c) and the relationships between social support and optimism and coping strategies will be mediated by stoicism.

We have also carried out additional statistical analyses (using SEM) to predict this model and have modified the last results section by adding a new text and two new figures:

*“Trajectory analysis results: relationship among stoicism and psychological variables*

*In men, as displayed in Figure 1, social support and optimism were directly, and negatively associated with stoicism (β =-0.17 and β =-0.23, respectively). Stoicism was directly, and positively associated with anxious preoccupation (β =0.12). Social support was positively associated with positive aptitude (β =0.17), and depression was positively associated with anxious preoccupation (β =0.56). The model fitted the data perfectly, X2=11.318, p=0.045; CFI=0.97; TLI=0.95; NFI=0.96; RMSEA=0.05 (90% CI [0.001, 0.074]).*

*In women, as displayed in Figure 2, stoicism was directly, and negatively associated with social support and positively with age (β =-0.21 and β =0.21, respectively). Optimism was directly, and negatively associated with stoicism (β =-0.14), and positively with anxious preoccupation and helplessness (β =0.26 and β =-0.30, respectively). Stoicism was directly, and positively associated with helplessness (β =0.12). The model fitted the data perfectly, X2= 14.447, p=0.044; CFI= 0.98; TLI= 0.95; NFI= 0.97M RMSEA= 0.04 (90% CI [0.000, 0.064]).”*

***Figure 1****. Tested study model: relationship between stoicism and psychological variables in men. Note: Dashed lines indicate statistically non-significant paths. *p<0.01; **p<0.001.*

***Figure 2****. Tested study model: relationship between stoicism and psychological variables in women.*

*Note: Dashed lines indicate statistically non-significant paths. *p<0.01; **p<0.001.*

**Reviewer #4:** This is an interesting study that will start an important conversation about the role of stocism in coping with cancer. There are several weaknesses that need to be addressed:
1. The introductory section does not really create the right background to the paper even though it was amended from the original. The authors need to present an argument that provides the basis for exploring stocism in cancer.

**Comment**: we have modified several sections of the introduction to present a robust and well-argued theoretical framework to justify the study of the influence of stoicism on psychological variables associated with coping with cancer.

At the end of paragraph 2 of the introduction we have added this text:

*“Some studies have shown that stoicism would be very sensitive to the health setting, and this could have implications for the patient's treatment and prognosis [6, 7]. Maintaining a stoic attitude towards pain and knowing one's own limits could be particularly beneficial for the patient and allow them to adopt a more adaptive coping style [4], or conversely, the stoic attitude could restrict attempts at medical intervention if the patient does not report their real clinical situation (i. e., the patient feels pain, but does not show it). Therefore, according to other researchers, stoic characteristics hinder seeking care, delay diagnosis, and prevent the implementation of improvements in psychological care [1].”*

At the end of the introduction, we have added a fourth paragraph:

*“Overall, the interrelationships between social support, optimism, stoicism, coping strategies and psychological distress remain to be further investigated. In the oncology field, little has been explored about the pathways through which stoicism influences coping in cancer patients. Some authors suggest that men and older people have higher levels of stoicism than women [2,17] or younger people [1]. Other authors have suggested that men and older adults are more stoic because they find it more difficult to identify and express their emotions [18], which would have a negative influence on seeking psychological help [19]. Meanwhile, other research suggests that the stoic attitude is more related to the ability to self-regulate one's emotions [12].”*

2. This is a substudy of a larger study I take it. That said the aims of the larger study are not articulated and there are no aims for this study or research questions that guided the analysis.

**Comment**: In response to this comment, we have included the following text with the hypotheses to make it easier to read and to better express what we expect to find:

*“In this perspective, our study has assessed the fit of an adapted stoicism model [4,5] (figure 1) using data collected from a large sample of patients who had cancer resected with curative intent. This adapted model of stoicism represents key constructs (social support, optimism, psychological distress, stoicism, and coping strategies), as well as the relationships between the variables used to operationalize the constructs. We presume that: a) social support and optimism will positively affect stoicism and coping strategies; b) stoicism will be related to coping strategies; and c) the relationships between social support, optimism and coping strategies will be mediated by stoicism.”*

3. The lack of an aim and research questions limits that discussion of findings and is a weakness. I was also disappointed that the discussion did not point to the potential clinical relevance of the construct, how you might assess in practice or point to the sort of research that is now needed if the construct has merit for further investigation.

**Comment**: We hope that the manuscript has been improved by the rewriting of the theoretical framework in the introduction, the inclusion of concise objectives and hypotheses, and explanations in the discussion that make clear the relevance, pertinence and applicability of this research. We have also included the prevalence of stoicism:

*“Overall, 27% of patients had low (PD≤ 46), and 33% had high stoicism scores (PD≥64).”*

I have also made some notes on the manuscript as at times the authors make comments that suggest a causal or directional relationship that the study is not set up to demonstrate.

**Comment**: we have included a new analysis with Structural Equation Modeling (SEM) to support the assertions we made in the manuscript and which we have explained in detail in the questions raised by reviewer #3. This is the text added to the statistical method section:

*“In this study, the previously determined significant factors were used in the SEM to identify the relationship between stoicism and psychological scales. Standardized direct, indirect, and total effects with corresponding 95% bias-corrected confidence intervals (CI) were measure using the bootstrapping methods [28,29]. The model fit was tested using the normed χ2 value (NC; desired value < 2.0, desired significance P > 0.05), the goodness-of-fit index; the Comparative Fit Index (CFI), the Tucker-Lewis index (TLI), the Normed Fit index (NFI) (>0.95 indicating an excellent fit); root mean square of approximation (RMSEA; desired value< 0.06) [28].”*

**Reviewer #5:**  The revised version is much better than the original one. I have only minor concerns related to some points that I listed below. The aim of this study should be the same in the abstract and at the end of the introduction.
It was stated in method that 932 individuals were recruited. It should be mentioned in methods how many participants were included and who many were male or female.

**Comment**: we have reviewed the objective of the abstract and the text and adjusted them to match. We have added how many patients were women and men through the following text (results section): “*Mean age was 62.1 years (standard deviation [SD]=12.1, range 24-85), 61% (n=566) were female and 39% (n=366) were male.”*

Cognizant of the contribution their insightful and critical observations have made to enhancing the quality of the text, the researchers of this study would again like to thank the Editor and reviewers for their time and effort.
